# Extending the use of biologics to mucous membranes by attachment of a binding domain

**DOI:** 10.1038/s42003-023-04801-6

**Published:** 2023-05-02

**Authors:** Robert M. Q. Shanks, Eric G. Romanowski, John E. Romanowski, Katherine Davoli, Nancy A. McNamara, Jes K. Klarlund

**Affiliations:** 1 Department of Ophthalmology, University of Pittsburgh School of Medicine, Pittsburgh, PA USA; 2 Charles T. Campbell Laboratory of Ophthalmic Microbiology, University of Pittsburgh, Pittsburgh, PA USA; 3 School of Optometry and Vision Science Graduate Program, University of California, Berkeley, CA USA

**Keywords:** Recombinant protein therapy, Protein delivery

## Abstract

Biologics are almost exclusively administered systemically, but localized delivery is preferable as it minimizes off-target exposure and allows more aggressive treatments. Topical application of biologics to epithelia is generally ineffective because most are covered with fluids and biologics are washed out too quickly to have significant therapeutic effects. Here we explore the idea that attaching a binding domain can serve as an “anchor” to extend the residency time of biologics on wet epithelia, allowing their effective use even with infrequent applications. We use topical application to the ocular surface as a challenging test since foreign substances are washed out especially efficiently by tear flow and blinking. Our results demonstrate that conjugation of antibodies to wheat germ agglutinin, which binds GlcNAc and sialic acid that are ubiquitously present in tissues, increases their half-life 350-fold upon application to the ocular surface in a mouse model of dry eye, a common and onerous disease in humans. Importantly, antibodies to IL-17A, IL-23, and IL-1β conjugated to the agglutinin reduces manifestations of dry eye, even when applied just once daily. In contrast, unconjugated antibodies are ineffective. Attaching an anchor to biologics is a simple means to overcome washout and to extend their therapeutic use.

## Abstract (web summary)

Conjugation of anti-cytokine antibodies to an anchor, wheat germ agglutinin, is demonstrated to be an effective method for increasing retention of biologics and allowing their therapeutic use on wet epithelia in an animal model of dry eye disease.

## Introduction

The wide-spread use of biologics is arguably among the most important advances in medicine in the last few decades. For instance, anti-inflammatory biologics have very significantly improved treatment of psoriasis, inflammatory bowel disease, and various forms of arthritis^1,2^. However, their use requires a delicate balance between achieving therapeutic effects and avoiding compromising the overall function of the immune system. Biologics are almost exclusively used systemically, but topical application would reduce side-effects and could be very beneficial in treating inflamed epithelia and surrounding tissues. However, potent mechanisms remove foreign substances effectively from such sites for instance by movement of cilia and flow of mucus and fluid, and topically applied biologics are normally removed too quickly to exert any significant effects unless they are applied extremely frequently.

**Fig. 1:**
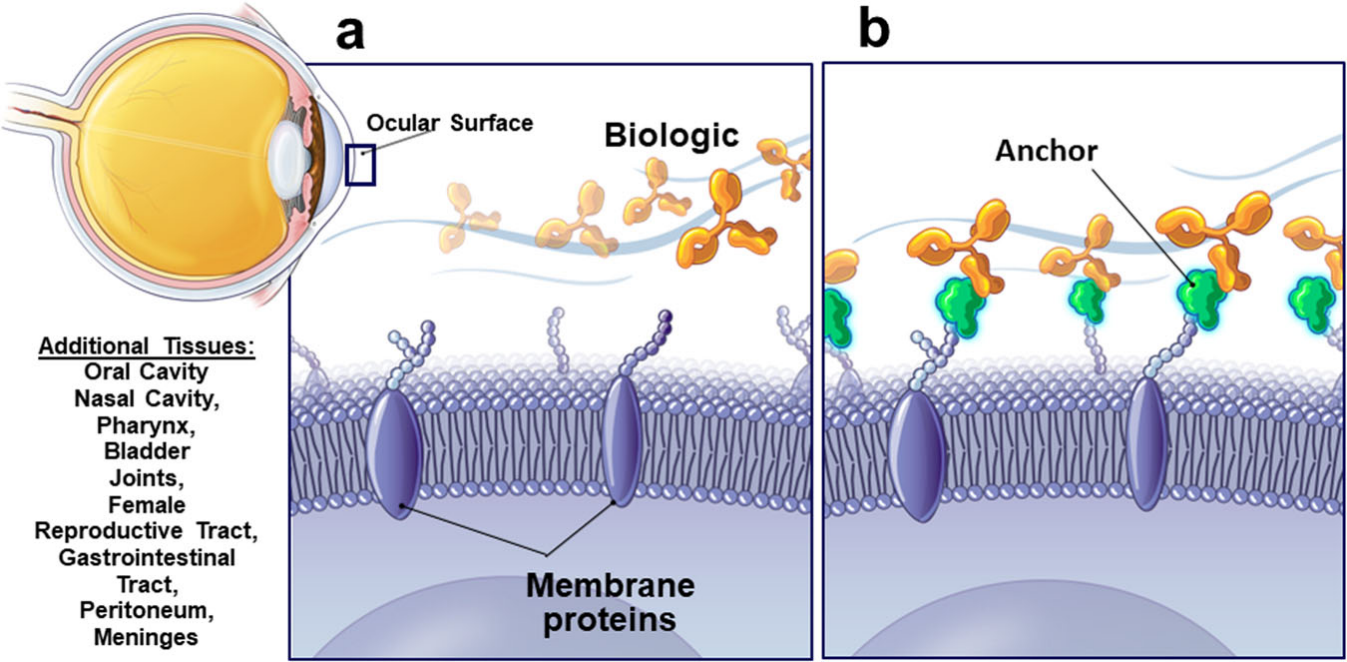
The anchoring concept. **a** Biologics, for example therapeutic antibodies, are rapidly washed out when applied to mucous membranes. **b** Attaching an anchor to the biologic that binds to the tissue immobilizes the biologic and allows it to act. Here we use wheat germ agglutinin (WGA) which binds to polysaccharides on cell surfaces.

The aim of this work was to test the concept that attaching a small module that binds to tissues will cause anchoring of biologics and allow them to act locally (Fig. 1). We used application to the eye, which has particularly efficient clearing mechanisms in the form of blinking and tear flow^3^. To evaluate this approach, we used a mouse model for dry eye. This is one of the most common ocular diseases that affects up to 50% of some populations and is more prevalent among women^4^. It ranges in severity from substantial annoyance to interfering with normal job functions^5,6^, and the condition is notoriously difficult to treat so new approaches are greatly needed^7,8^. Dry eye is initiated by hyperosmotic stress of the corneal epithelium and conjunctiva, which results in an inflammatory response that induces extensive damage to the ocular surface^9–11^. In this study we examined the effects of antibodies to major inflammatory cytokines using wheat germ agglutinin (WGA) as an anchor. WGA binds to N-acetyl glucosamine and sialic acid^12^ which are ubiquitous components of polysaccharides in mucins, on the cell surface, and in extracellular matrix. There are therefore numerous binding sites for WGA in tissues including the ocular surface^13,14^. Domains of other proteins that bind to extracellular matrix have been used to immobilize biologics after injection into tissues^15,16^. Here, we examine whether application of WGA-conjugated antibodies allow their use on mucous membranes as exemplified by the ocular surface.

## Results

### Antibodies conjugated to WGA retain their ability to bind antigen

**Fig. 2:**
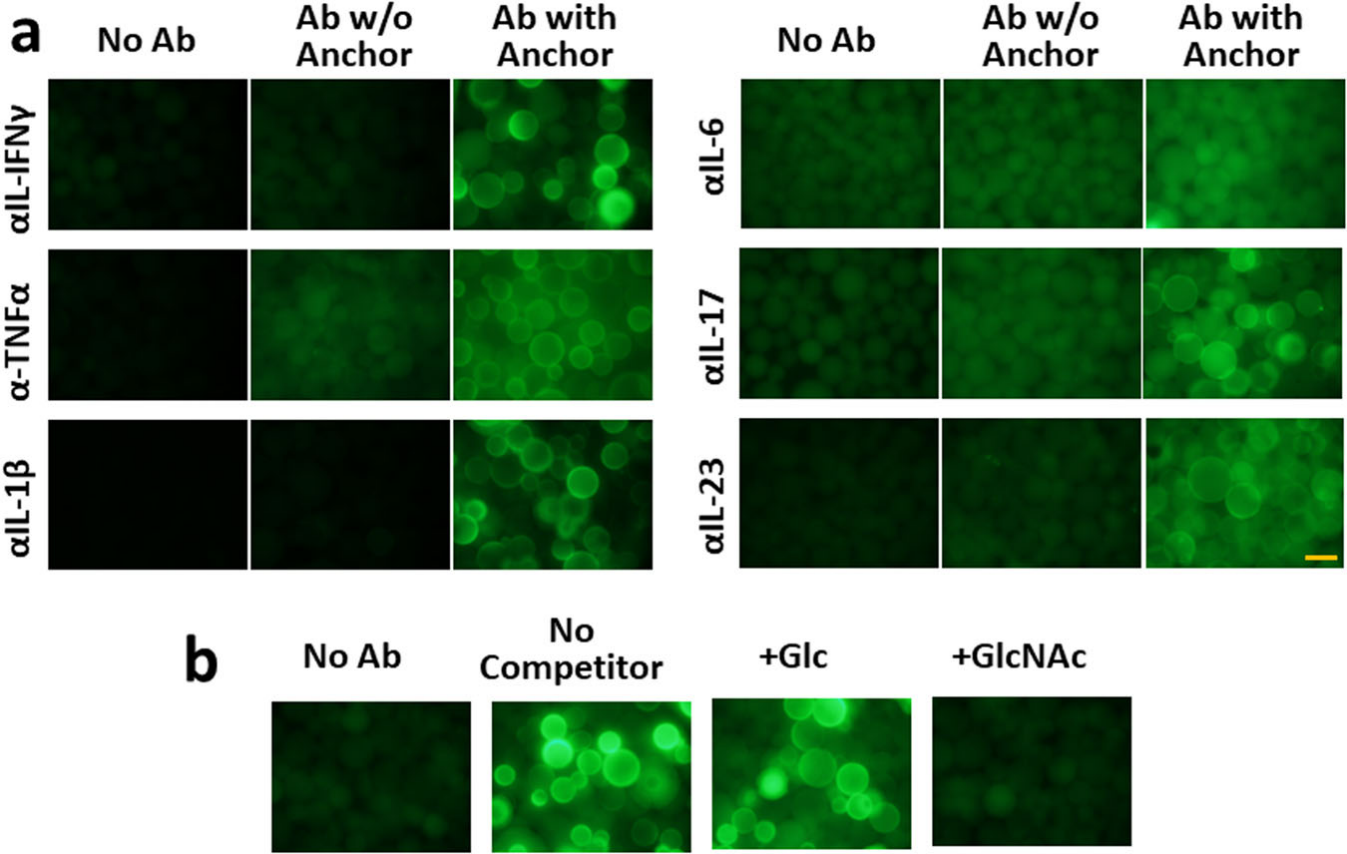
Retention of binding capacities of antibodies after conjugation to WGA. **a** Binding of ligands to WGA-conjugated antibodies. GlcNAc agarose beads were incubated with the indicated antibodies and subsequently with fluorescently labeled ligands and photographed with a fluorescence microscope. Binding does not occur to the WGA anchor (cf. Supplementary Fig. 4). Quadruplicate values ± SE. **b** Specificity of binding to GlcNAc. Anti-IL-1β antibodies were incubated with GlcNAc agarose beads in the presence of 0.2 M of the indicated competitors. After washing, they were incubated with fluorescently labeled IL-1β as indicated. Similar experiments with additional antibody conjugates are shown in Supplementary Fig. 2. The orange scale bar represents 0.1 cm.

Antibodies were conjugated covalently to WGA using standard cross-linking agents ((1-ethyl-3-(3-dimethylaminopropyl) carbodiimide hydrochloride and N-hydroxysulfosuccinimide)^17^, and successful cross-linking was verified by appearance of new bands upon SDS-PAGE (Supplementary Fig. 1). WGA binds GlcNAc and sialic acid^12^, and the conjugates are expected to contain binding sites to both GlcNAc and to the cognate antigens. To test this, the conjugates were incubated with GlcNAc-coated agarose beads, washed, and incubated with fluorescently labeled ligands. WGA-conjugated antibodies to all six tested cytokines were capable of binding the ligands to the beads (Fig. 2a). Furthermore, a competition assay confirmed that the conjugates bind to GlcNAc (Fig. 2b). This shows that both the binding sites of WGA for GlcNAc and the binding sites of the antibodies for their ligands remain functional. To assess the efficacy of conjugation, pull-down experiments were performed with GlcNAc agarose beads, which showed that 38-55% of the protein in the preparations bind to the beads (Supplementary Fig. 3). The conjugates retain their specificities of binding to their cognate ligands (Supplementary Fig. 4), and have similar ligand binding affinities (Supplementary Fig. 5)

### Conjugating antibodies to WGA increases their residency time on the cornea

**Fig. 3:**
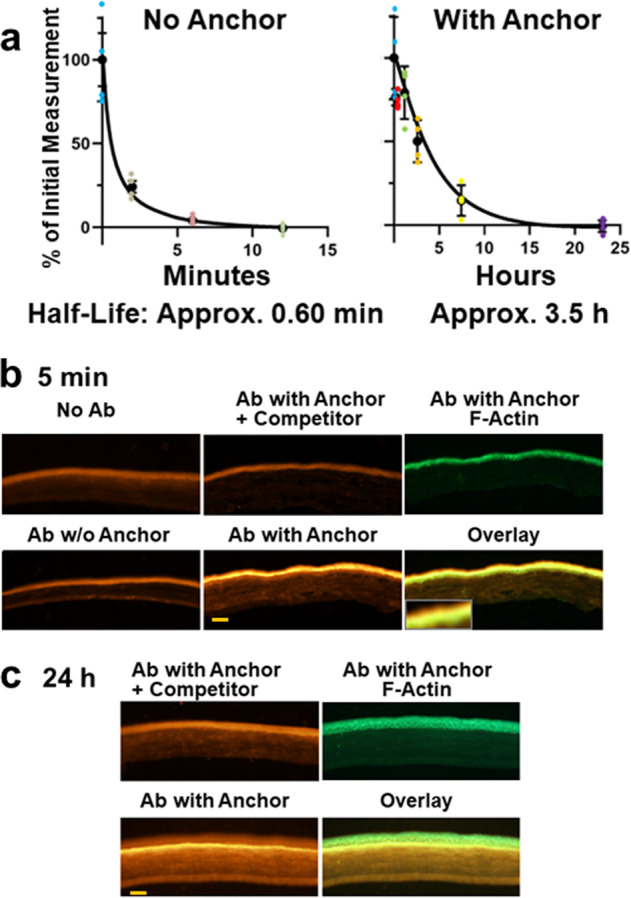
Retention of IL-17A antibody on the ocular surface of mice operated to induce dry eye. **a** Retention of fluorescently labeled IL-17A antibody. Alexa Fluor 488-labeled antibodies were applied to eyes and followed by fluorescence imaging. Quadruplicate determinations, means ± SE. **b**, **c** Histological analysis of applied antibody (Ab) in tissues. The anti-IL-17A antibody was raised in a rat host and was detected with an Alexa Fluor 555 labeled anti-rat IgG antibody (bright orange) (**b**) IL-17A antibody was applied to eyes and they were harvested 5 min later. The eyes were treated with no antibody, antibody without anchor, or antibody with anchor, as indicated. The specificity of the signals was verified by preincubating the anti-rat IgG with the anti-IL-17A antibody (“+Competitor”). The epithelium was visualized by staining with phalloidin, which stains f-actin (green). The overlay is a composite of the anti-IL-17A antibody with anchor condition stained with anti-IgG and with phalloidin, and the insert in is a higher magnification of a section of the epithelium showing presence of antibody in the mucin layer (yellow). **c** As (**b**) except the mice were harvested 24 h after application of anti-IL17A antibody and a longer exposure time for photography was used. Antibody is seen in the lower part of the epithelium and in the anterior part of the underlying stroma in some regions. The orange scale bars represent 100 µm.

Removal of the major extraorbital lacrimal gland induces severe aqueous deficient dry eye and is the basis for a well-characterized animal model^18–20^. We analyzed retention of an antibody to IL-17A because intraperitoneal injection of antibodies to IL-17A has been shown to reduce dry eye signs in mouse models^21–23^. The antibody was labeled with Alexa Fluor 488, and the presence on the ocular surface was followed by fluorescence photography. As expected, free antibody was removed rapidly from the eye with a half-life of 30 s (Fig. 3a)^24,25^. Importantly, the half-life of the antibody conjugated to WGA complexes was 350-fold higher. To verify retention of the complexes, the anti-IL-17A antibody was followed by immunohistochemistry. Five minutes after application, unconjugated antibody was not detected, as expected from its rapid washout. In marked contrast, antibody conjugated to WGA was seen prominently throughout the epithelium and in the mucin layer, which is very extensive on the ocular surface^26^ (Fig. 3b). After 24 h, antibody was detected in the lower parts of the epithelium and in the underlying basement membrane, which contains abundant binding sites for WGA^27^, in some regions (Fig. 3c). The antibody was absent after 72 h. Together, these data demonstrate a very marked increase of retention of the antibody upon conjugation to WGA.

### Application of anchored anti-IL-17A antibody once a day is sufficient to treat dry eye

**Fig. 4:**
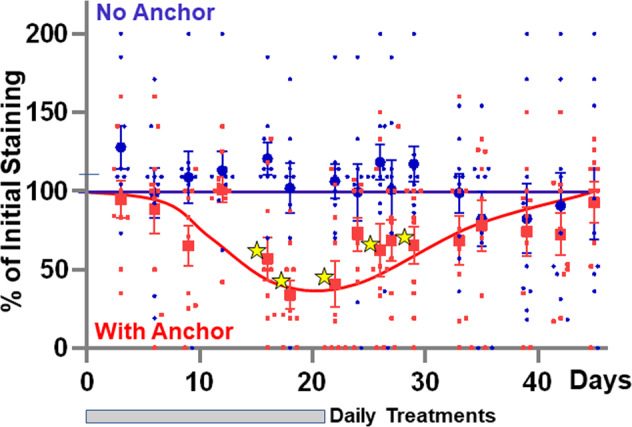
Effect of anchored IL-17A antibodies on fluorescein staining of eyes. Dry eye was induced by surgical removal of the major lacrimal gland and treated daily with anti-IL-17A antibody with or without anchor, as indicated. The eyes were stained with fluorescein and levels of staining scored. 9 eyes were in the “No Anchor group” and 10 eyes in “With Anchor” group. The stars indicate statistical significant reduction in staining (*p* = 0.0025, 0.0013, 0.0021, 0.015, and 0.0060 after 16, 18, 24, 26, and 29 days, respectively). Values are means ± SE. The “No Anchor” group had 9 samples, the “With Anchor” had 10 values.

Fluorescein staining of the cornea reflects damage to the epithelium and is widely used as a clinical test for dry eye in humans^28^. Strong fluorescein staining was evident after removal of the lacrimal glands from the mice and, importantly, a reduction in fluorescein staining was evident starting 8–12 days after once-daily application of WGA-conjugated anti-IL-17A antibodies (Fig. 4). Anti-IL-17A antibodies without the WGA anchor were without effect as expected from the rapid washout (Fig. 3). When treatment was discontinued (Day 21), the fluorescein staining reverted to untreated levels after 7–10 days (Fig. 4), indicating a requirement for continuous presence of the antibody to reduce damage to the cornea.

**Fig. 5:**
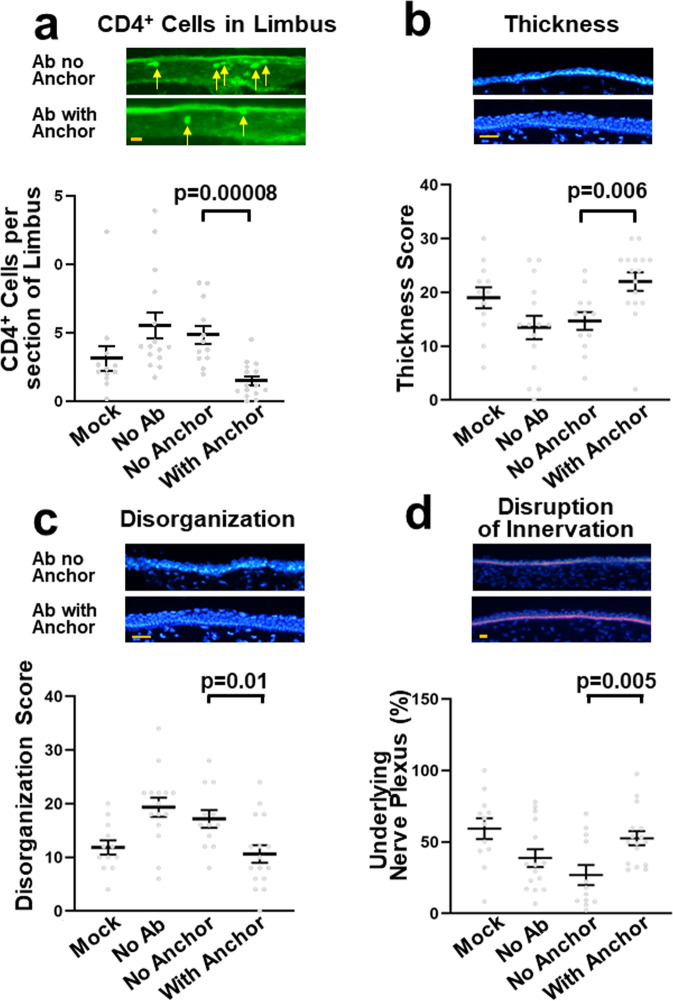
Histological analysis of the effects of anchored anti-IL-17A. The mice were treated as indicated on the x-axis. Corneas were evaluated by histology for (**a**) presence of CD4^+^ cells in the limbus (arrows), (**b**) epithelial thickness, cell nuclei were visualized with the Hoechst 33342 stain, (**c**) degree of disorganization of the normal layering into basal, wing, and squamous cells (Supplementary Fig. 7), and (**d**) the extent of the epithelium that was associated with underlying basal nerve plexus as detected with an anti β3-tubulin antibody (red), while the nuclei were detected with the Hoechst stain (Blue). In (**b**, **c**) the sum of scores from two masked observers are shown. The key comparison of treatment without and with anchor yielded significant results in all cases (Student t test). In contrast, treatments with antibody without anchor did not result in any significant changes in the extent of pathology of any of the parameters (“No Antibody” compared to “No Anchor”) (*p* > 0.20). The number of samples in the groups are: Mock, 12; No Ab, 15; No Anchor, 12; With Anchor 16. The data are pooled from two identical experiments harvested 24 and 26 days after treatment. Orange scale bars (yellow) represent 50 µm. The number of measurements were (Mock, No Ab, No anchor, with anchor): CD4+ cells, 12, 15, 12, 15: Thickness, 12, 15, 12, 16: Disorganization, 12, 15, 12, 16: and Innervation, 12, 15, 12, 16. Error bars are SE.

Additional manifestations of dry eye^9,29–31^ were monitored in order to confirm the efficacy of the treatments. Very few CD4^+^ cells were present in the central cornea in both control and operated mice. CD4^+^ cells were evident in the limbus (a vascularized structure surrounding the cornea), and their numbers were significantly increased after induction of dry eye in accord with the inflammatory nature of the disease^9–11^. The increase was prevented by treatment with WGA-conjugated antibodies (Fig. 5a). In contrast, unconjugated antibodies were ineffective, as expected from their rapid washout. Lacrimal gland removal induced significant epitheliopathy: the corneal epithelium was thinner and lost the characteristic three-layered organization into basal, large wing, and flat squamous cells. These changes were reversed by treating with anchored, but not with non-anchored, anti-IL-17A antibodies (Fig. 5b, c). A sub-epithelial nerve plexus underlies the corneal epithelium, and it was partially lost upon lacrimal gland removal. Treatment with WGA-conjugated antibodies rescued the loss of the sub-epithelium nerve plexus by treatment with anchored, but not with non-anchored antibodies (Fig. 5d). This analysis confirms that anchoring is an effective means to convert the anti-IL-17A antibody into an effective drug that acts even when applied just once daily.

### Anchored anti-IL-23 and IL1β antibodies are also effective to treat dry eye

**Fig. 6:**
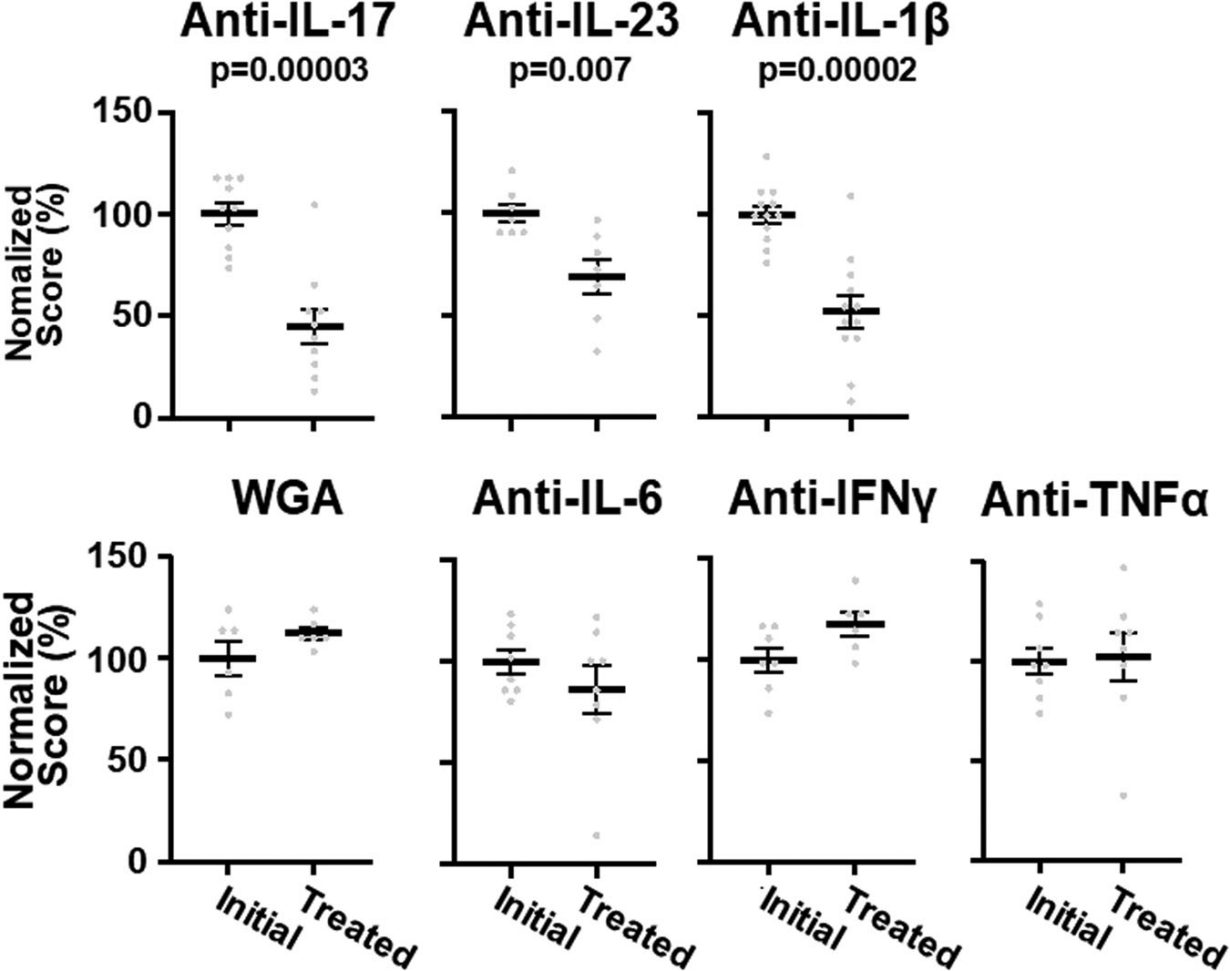
Effects of anti-cytokine antibodies on dry eye. Eyes were treated once daily with antibodies to the indicated cytokines for 3–4 weeks. The eyes were scored by fluorescein staining before and after treatment. Experiments were done 2–6 times with similar results. The group sizes were: anti-IL-17A, 10; anti-IL-23, 7; anti-IL1β,12; WGA, 6; anti-IL-6, 8; anti-IFN, 7; and anti-TNFα, 8. Values are means ± SE.

Numerous cytokines have been described to have roles in generation of dry eye^9–11^, and we tested the effects of antibodies to additional targets on fluorescein staining, similarly to the experiments shown in Fig. 4. Anchored antibodies to IL-23 were effective, which was anticipated because IL-23 has an important role in maintaining the Th17 cell population^32^ (Fig. 6). Anchored antibodies against IL-1β also reduced fluorescein staining in accord with reports that antagonists to IL-1β are effective in treating dry eye^33^. In contrast, no significant reduction in fluorescein staining was seen after treatment with anchored antibodies to IL-6, TNFα, IFNγ, or with the WGA anchor alone.

## Discussion

Despite their great potency, biologics are rarely used topically at mucosal surfaces in the body because they are washed out too quickly to have therapeutic effects. Only one biologic, Oxervate^TM^, is presently approved by the US Food and Drug Administration for topical use in humans^34^, and it must be applied six times per day. The results presented here show that the simple strategy of attaching a WGA anchor increases the half-life of residency on the ocular surface 350-fold. This is different from traditional delivery systems that are based on some form of carrier. Carriers can be gels, pellets, contact lenses, or various forms of microparticles that may cause irritation and inflammation, provide substrata for bacterial growth, fall out, and/or interfere with vision or other functions of the eye and other tissues^35,36^. Eliminating the carrier circumvents these problems and allows biologics to be delivered in eye drops, mouth washes, and other familiar types of liquid formulations.

Many biologics are directed to curb inflammation and here we demonstrate the usefulness of antibodies to cytokines to treat dry eye, which is a very common and onerous condition that is driven by inflammation of the ocular surface^9–11^. Application of a WGA-conjugated antibody to IL-17A just once daily is sufficient to treat dry eye in a mouse model whereas the unconjugated antibody is ineffective. A role for IL-17A in dry eye has been suggested by its increased levels at the ocular surface in animal models and in humans^37,38^. Furthermore, in vivo neutralization of IL-17A ameliorates dry eye and adaptive transfer of CD4^+^ IL-17A producing cells induces the disease in naïve mice in several models^21–23,38–40^. IL-1β is an important inducer of inflammation, and, similarly to IL-17A, causes release of numerous cytokines and breakdown of epithelial barrier function^41–43^. Blocking IL-1 signaling has been shown to counter dry eye in animal models and clinical trials have suggested an effect of blocking IL-1 in humans^31,33,44,45^. In contrast, antibodies to IL-6, TNFα, and IFNγ were ineffective in our model. Some caution should be used in interpreting these negative data. The situation in vivo is complex, and the localization and kinetics of production of endogenous cytokines could preclude efficient neutralization by the antibodies. Dry eye in humans is a highly heterogenous disease, and IL-6, TNFα, and IFNγ have all been implicated in the disease^9^, and there are many models for dry eye featuring different pathophysiologies and different mechanisms of induction of inflammation^19,20^. It remains undertermined whether IL-6, TNFα, and IFNγ may have roles in the model used here.

The present approach to drug delivery is based on rapid binding kinetics and presence of plentiful binding sites. In this study, we therefore used WGA, which binds sialic acid and GlcNAc that are abundant extracellularly in tissues^12^. The abundance of binding sites is also the basis for use of WGA and other lectins experimentally as a mucoadhesive to attach liposomes and other microcarriers to epithelial surfaces^46^. A high concentration of WGA (10%) was found to exhibit no evident toxic effects in rabbit and human eyes, and a 5% solution of fluorescein-labeled WGA was recently determined to have no detrimental effects on visual acuity and was suggested as a routine tool to detect dry eye^13,47^. Heparan sulfate is another possible target, and we have previously shown that a brief exposure to heparan sulfate binding EGF-like growth factor is sufficient to induce migration of corneal epithelial cells in vitro^48^. Collagen is a major component of connective tissues and collagen binding domains can be used to immobilize drugs after injection into tissues^16^. In the eye, we have shown that collagen binding domains can target chimeric proteins to lesions in the ocular surface when applied topically^49^, and they could be useful to direct pharmaceuticals to regions of epithelial breakdown. Attachment was done here by chemical cross-linking, which inevitably yields heterogenous products, for instance by attaching at different sites in the antibody molecules or by linking multiple anchors to the antibodies. A future direction could be to attach anchor domains by genetic engineering, yielding uniform products.

Intact epithelium constitutes a significant barrier to delivery of large molecular weight drugs such as protein biologics^50^. In the present model, the epithelium is damaged as demonstrated by the strong fluorescein staining and the histological analysis (Fig. 5). Epithelial damage is a hallmark of dry eye disease in humans^28^, and damage is common in diseases of the ocular surface, which is presumably the basis for the numerous reports that application of protein biologics can have therapeutic effects in the cornea in both animal models and in humans when applied sufficiently frequently^33,34,45,51–57^. Damage allows the applied WGA-conjugated antibody to penetrate into the epithelium and accounts for its presence under the epithelium in some regions (Fig. 3c). Disruption of the epithelial barrier function is also expected to facilitate diffusion of inflammatory cytokines so they can be neutralized by antibodies located either in the epithelium or in the mucin layer on the ocular surface.

Chemical cross-linking is a universal procedure that can be used to couple to any biologic to WGA, but the near-random nature of the reactions results in heterogenous products. The preparations used here contain some unconjugated antibody (Supplementary Fig. 3). This, however, is washed out too quickly to have any detectable effects (Figs. 3, 4). Likewise, any residual WGA would be inactive in the fluorescein assays (Fig. 6). The conjugates generated here should be considered lead compounds that need further refinement before any human trials are contemplated. Final drugs could be, for instance, genetically engineered antibodies or derivatives fused to anchoring domains. Such constructs would have a totally defined polypeptide, and any variability would arise from post-translational processing, as with other antibody biologics.

Most epithelia are covered by fluids and have various forms of clearance mechanisms of foreign substances. This includes, for instance, the cornea, the oral cavity, joints, the bladder, the GI tract, the lungs, the female reproductive system, the peritoneum, and the meninges. It is clearly preferable to use biologics locally to minimize side-effects and to allow higher local concentrations. Successful treatment with anti-inflammatory biologics requires a delicate balance of maintaining the overall efficacy of the immune system while reducing inflammation at affected sites only, a balance that is difficult to achieve when they are used systemically^58–60^. Importantly, biologics attached to a suitable anchor is expected to only require application once or twice daily which is a realistic regimen for patients, and this approach is therefore expected to expand the usefulness of biologics.

## Methods

### Conjugation of WGA to antibodies

400 µL WGA, 1 mg/ml, in buffer 1 (50 mM 2-(N-morpholino) ethanesulfonic acid, 500 mM NaCl, pH 6.0) was placed in a 10 kDa Amicon^®^ 500 µL centrifugal filter unit, and activated by adding 10 µL freshly prepared 80 mM 1-ethyl-3-(3-dimethylaminopropyl) carbodiimide hydrochloride and 10 µL 200 mM N-hydroxysulfosuccinimide in water and incubated for 10 min at room temperature. The filter unit was centrifuged 3 times for 15 min at 15,000 × *g* with 100 µL buffer 1 diluted 10 times with water. 250 µg antibody was concentrated in a separate 100 kDa Amicon centrifugal filter unit and washed 3 times in 0.1 M HEPES. pH 7.5 at 15,000 × *g* for 15 min. The concentrated and activated WGA was added to the antibody, and incubated at 4 °C overnight. The reaction was quenched by addition of 10 µL 100 mM ethanolamine. An unconjugated antibody preparation was treated identically except no WGA was added. The preparations were washed and transferred to corneal application buffer (10 mM HEPES, pH 7.2, 132 mM NaCl, 24 mM KCl) and free WGA (MW 38 kDa) was removed by three centrifugations with 200 µL of the buffer in the Amicon 100 kDa cutoff filter units. Protein was measured with the Pierce Coomassie Protein Assay Kit and adjusted to 1 µg/µL (0.75 µg/µL in some experiments). The efficacies of the preparations to bind cytokines to GlcNAc were routinely verified as described below. For analysis by gel-electrophoresis, protein samples were mixed in equal volumes with SDS gel-loading buffer (100 mM Tris-Cl, pH 6.8, 200 mM dithiothreitol, 4% SDS, 0.2% bromophenol blue, and 20% glycerol) and placed in a heating bock at 100 °C for 10 minutes, cooled on ice for 1 minute, and centrifuged at 10,000 × *g* for 1 minute. Samples were loaded into PAGE gels (10–20% BioRad Mini-PROTEAN Tris/Tricine) and separated using Tris-glycine-SDA buffer (BioRad 1610732). After separation, the gels were stained with Coomassie Brilliant Blue (EZ-Run protein gel staining solution, Fisher Scientific). To determine conjugation efficiency, 10 µL GlcNAc beads (Sigma-Aldrich) were washed with HBS (20 mM HEPES, pH 7.2, 100 mM NaCl), supernatants carefully aspirated, and 4 µL of the conjugate added. After overnight incubation, 25 µL HBS was added, the beads agitated and spun down at 15,000 g for 30 s. 20 µL of the supernatant was collected for assay with the BCA reagent (Pierce^TM^) (unbound fraction). After three washes with 200 µL HBS and careful aspiration of supernatant, bound protein was eluted with 29 µL 1% SDS, the beads spun down as before, and 20 µL collected for protein assay (bound fraction). The neutralizing antibodies used in these studies were: anti-IL-1β, clone 1400.24.17 (Invitrogen); anti-IL-6, clone MP5 20F3 (Invitrogen); anti-IL-17A, clone TC11-18H10 (BD Biosciences); anti-IL-23, clone G23-8 (Invitrogen); anti-IL-IFNγ, clone H22 (R&D Systems); anti-TNFα, clone XT22 (Invitrogen).

### Labeling of ligand and testing binding to antibodies

10–100 µg of the ligands (PeproTech, Sino Biologicals) in 10 µL 0,1 M HEPES pH 7.5 were added to 40 µg Alexa Fluor 488 carboxylic acid, 2,3,5,6-tetrafluorophenyl ester (Molecular Probes) in a dry form and the reactions were allowed to proceed overnight at 4 °C. 10 µL 100 mM ethanolamine was added to quench the reactions and the volume was adjusted to 100 µL with HBS. To test binding activity qualitatively (Fig. 2) of the antibody conjugates, approximately 10 μL GlcNAc agarose beads were washed twice with HBS in Eppendorf tubes, the supernatants carefully aspirated, and 2 μL of the antibody conjugate was added, and the tube incubated for >1 h at room temperature with occasional agitation. The beads were washed three times with 200 μL HBS, the supernatants carefully aspirated, and 4 µL of the labeled ligands were added and incubated overnight with occasional agitation at 4 ^o^C. In some experiments, 0.5% Triton X-100 and 1% bovine serum albumin was added to reduce background binding. After five washes with HBS, aliquots of the beads were transferred to microscope slides and photographed with a 20X objective using a fluorescence microscope. To titrate binding of ligand (Supplementary Fig. 5), WGA-conjugated antibody was bound to GlcNAc or protein G beads. Binding of Alexa Fluor 488 ligand was assessed as described above and quantitated using ImageJ software^61^. The WGA/antibody-coated beads were incubated with the same amounts of Alexa Fluor 488 labeled ligand, increasing the volume in 2.5 fold steps, and incubating overnight under constant agitation.

### Removal of lacrimal glands

Mice were housed in a pathogen-free animal facility at the University of Pittsburgh and all procedures involving live animals were approved by the Institutional Animal Care and Use Committee (IACUC) (IACUC Protocol #17060565). All experiments conformed to the Association for Research in Vision and Ophthalmology (ARVO) Statement for the Use of Animals in Ophthalmic and Vision Research. 8–10 week old female C57BL/6 mice (Charles River Laboratories) were anesthetized with ketamine (100 mg/kg) and xylazine (10 mg/kg) injected intraperitoneally, and ketoprofen (5 mg/kg subcutaneously) was also administered subcutaneously at that time. The major extraorbital lacrimal glands of both eyes were excised as described^18^ except that a sterile razor blade was used to make the incision and surgical glue (Vetbond Tissue Adhesive, 3M Corporation), was used to close the wounds. Ophthalmic triple antibiotic ointment (Bausch and Lomb) was added daily for several days to prevent formation of bacterial keratitis. The dry eye phenotype was allowed to stabilize for at least 4 weeks before initiation of treatments. The operation resulted in 90% reduction of tear volume as assessed by measurement with Zone-Quick^TM^ phenol red cotton threads applied to the ocular surface in the lateral canthus for 30 s^18,62^.

### Application of therapeutics, and evaluation of treatments and retention

0.5 µL protein solution was applied gently to the surface of the eyes with a Gilson^®^ 2 µL pipette starting at least 4 weeks after the operation. Fluorescein staining was performed by applying 1.5 µL 0.2% fluorescein (Akorn Pharmaceuticals) in corneal application buffer and washing with 6 µL application buffer after 1–2 minutes. Pictures were taken with a camera fitted with a Wratten #12 filter, illuminating with a light source equipped with a Wratten #47B filter every 1–4 days. Photography was done at the same time of day to avoid circadian effects. Pictures of eyes were scored by two masked observers using a 0–3 calibrated scale (Supplementary Fig. 6). To define the baseline for the fluorescein staining, four consecutively taken pictures of each eye just prior to initiation of treatments were scored and the sum of the scores were used. The operation to induce dry eye was not successful in all cases and only eyes having a sum of scores of 12 or more in the four measurements were used for analysis. The mice were treated for 20–28 days and the sum of the scores of the last three determinations were used as measure of final outcome. This resulted in 25 possible values for determining the baseline and 19 possible values for the outcome, and they were converted to a common 0–100 scale for comparison. ANOVA or the two-tailed Student *t* test were considered appropriate and used for all analyses^63–65^, except for the disorganization score below for which a one-tailed test was used (since only decreased organization is possible). GraphPad Software was used for calculations. To measure retention, 250 µg anti-IL-17A antibody was labeled with Alexa Fluor 488 and conjugated to WGA as described above for labeling cytokines. 25 µg was applied to each eye and they were photographed at various times after application. Retention of unlabeled antibody was analyzed similarly, omitting the Alexa Fluor labeling step, and histological analysis was performed as described below. The anti-IL-17 antibody was raised in a rat and was detected with Alexa Fluor 555 labeled anti-rat-IgG (Invitrogen). For competition, 40 µg anti-IgG was preincubated with 25 µg anti-IL-17 in 1 mL total volume for 30 min before application to the slides.

### Histology

Mice were sacrificed by CO_2_ inhalation in accordance with the 2020 American Veterinary Medical Association (AVMA) Euthanasia Guidelines. Cervical dislocation was then performed to ensure death as required by the University of Pittsburgh IACUC. Eyeballs and surrounding tissue were removed and snap-frozen in optimal cutting temperature (OCT) compound (Fisher Scientific). 20 µm sections were cut with a Leica CM3050S Cryostat and applied to silane-coated slides (Azer Scientific). The sections were stained by first blocking with 20% non-immune goat serum in HBS, washed 3 times with HBS, and incubated with antibodies to CD4 (clone GK1.5) or to beta tubulin III (clone 2G10-TB3) pre-labeled with eFlour660 (Invitrogen) in 10% non-immune mouse and 10% non-immune rat serum in HBS for 1 h. The slides were then washed three times with HBS, and incubated with 0.25 µg/mL Hoechst 33342 stain and 0.6 U/mL Alexa Fluor 488 Phalloidin (Molecular Probes) for 3 min, washed three times, and coverslips applied using Shandon^TM^ Immu-Mount^TM^ mounting medium. Areas containing the limbus were photographed with an Olympus IX83 fluorescence microscope using a 20X objective and Dimension cellSens software^TM^, and the number of CD4^+^ cells were counted. Three fields were used for each eye. For assessing the disorganization and thickness, three fields of the cornea spanning the entire central region were photographed with a 20X objective and the disorganization and thickness of the epithelium in each field were scored by two masked observers on scales 0–3, and the sum of scores used (for scoring criteria see Supplementary Fig. 7). To measure the percentage of epithelium associated with underlying nerve plexus, the lengths of detectable plexus and epithelium in three fields spanning the central region of the cornea were measured with a Scale Master^TM^ opisometer.

### Statistics and reproducibility

Mean ± SE. values were used and were calculated using Prism version 9 software (GraphPad Software) using distinct biological samples, and two-tailed Student’ t test was used for comparison of groups. A *p* value of <0.05 was deemed significant. During establishment and optimization of the conjugation procedure and the animal model >10 experiments were performed to confirm enhanced retention of antibodies that all showed large increases of retention upon attachment of WGA. Histological localization of applied anchored vs. unanchored was performed >5 experiments with consistent results. Efficacy studies were performed at least twice with each of the tested antibodies, with consistent results, and results in the Supplementary Information was similarly performed twice with consistent outcomes. The data on histological markers (Fig. 5) were compiled from two separate experiments.

### Reporting summary

Further information on research design is available in the Nature Portfolio Reporting Summary linked to this article.

## Supplementary information

Klarlund_Peer Review File

Supplementary Information

Description of Additional Supplementary Files

Supplementary Data 1

Reporting Summary

## Data Availability

Data underlying the figures are in the Supplementary Data 1. The original stained gel is included in Supplementary Fig. 1. Other data generated and analyzed during the current study are available from the corresponding author upon reasonable request.
