# Supplementary material for: Extending the use of biologics to mucous membranes by attachment of a binding domain
Source: Commun Biol. 2023 May 2;6:477. doi: 10.1038/s42003-023-04801-6 (PMC10154311; doi:10.1038/s42003-023-04801-6)
Supplement: Supplementary file 2 — Supplementary Information [file 42003_2023_4801_MOESM2_ESM.pdf]

SUPPLEMENTARY INFORMATION

EXTENDING THE USE OF BIOLOGICS TO MUCOUS MEMBRANES BY ATTACHMENT OF A BINDING DOMAIN

Robert M. Q. Shanks, Eric G. Romanowski, John E. Romanowski, Katherine Davoli, Nancy A. McNamara, and Jes K. Klarlund

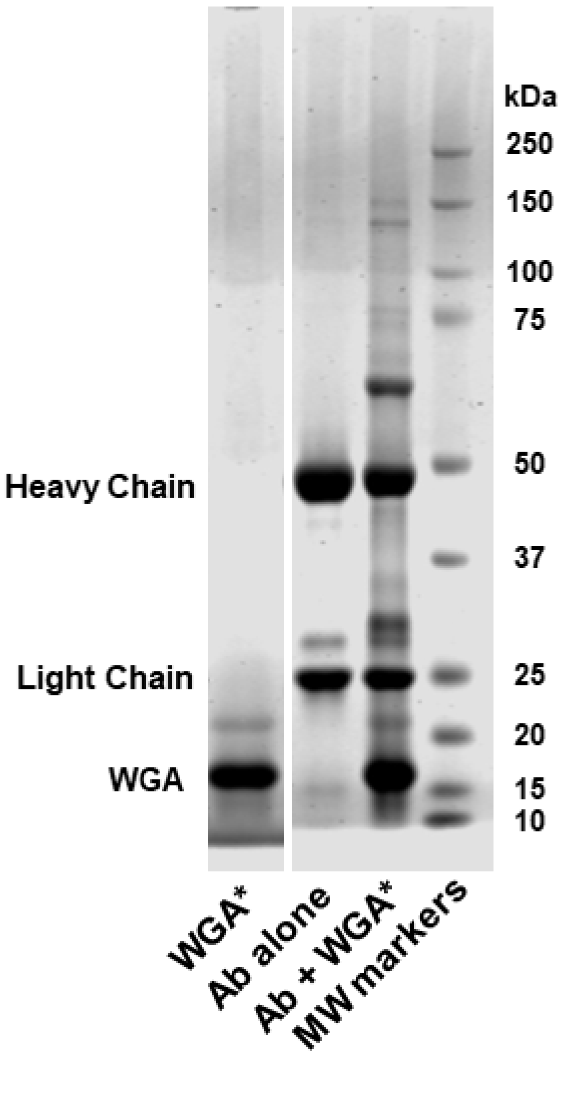

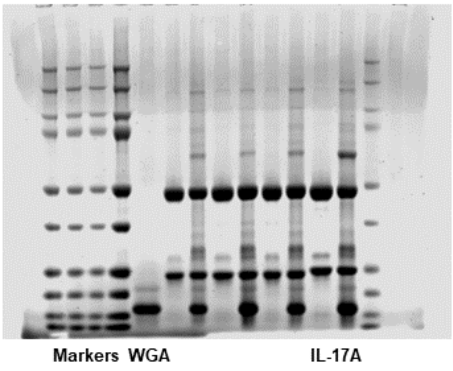

Supplementary Fig. 1. Appearance of covalently linked species after conjugation of anti-IL-17 antibody to WGA activated with (1-ethyl-3-(3-dimethylaminopropyl)carbodiimide hydrochloride and hydroxysulfosuccinimide. Preparations were analyzed by reducing SDS-PAGE. WGA* is WGA activated with the cross-linking agents. Right figure shows the original, uncropped, and unmodified gel.

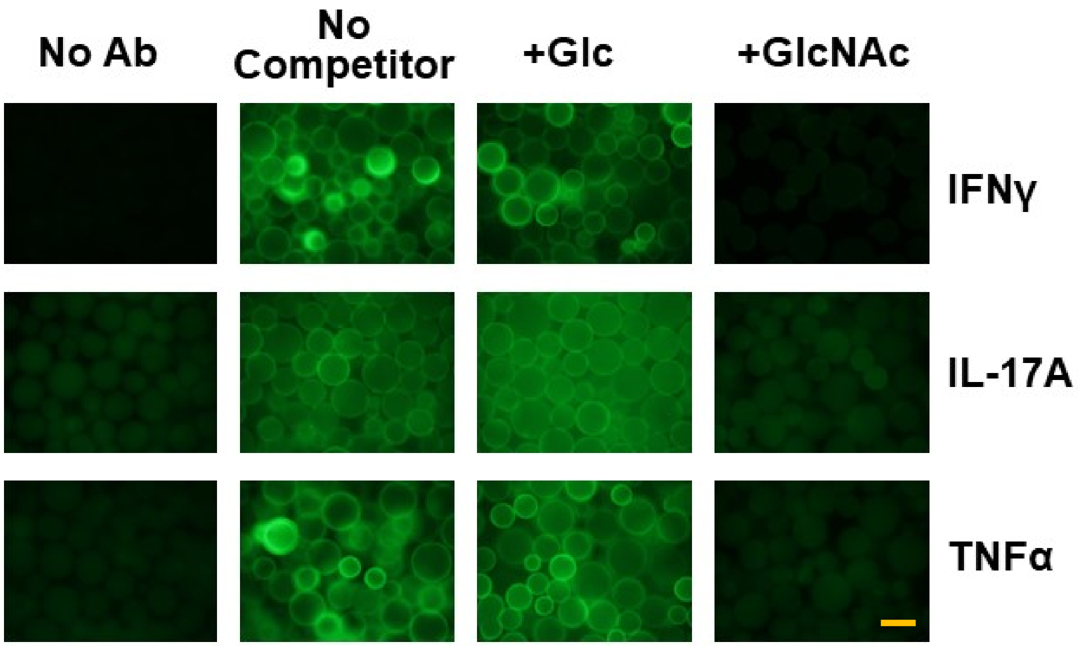

Supplementary Fig. 2. Specificity of binding of additional WGA-antibody complexes to GlcNAc (cf. Fig. 2b in the main text). The experiments were performed with the indicated cytokines and WGA conjugated anti-cytokine antibodies as described in Fig. 2b, except that 0.4 M of the competitors were used. The orange scale bar represents 0.1 cm.

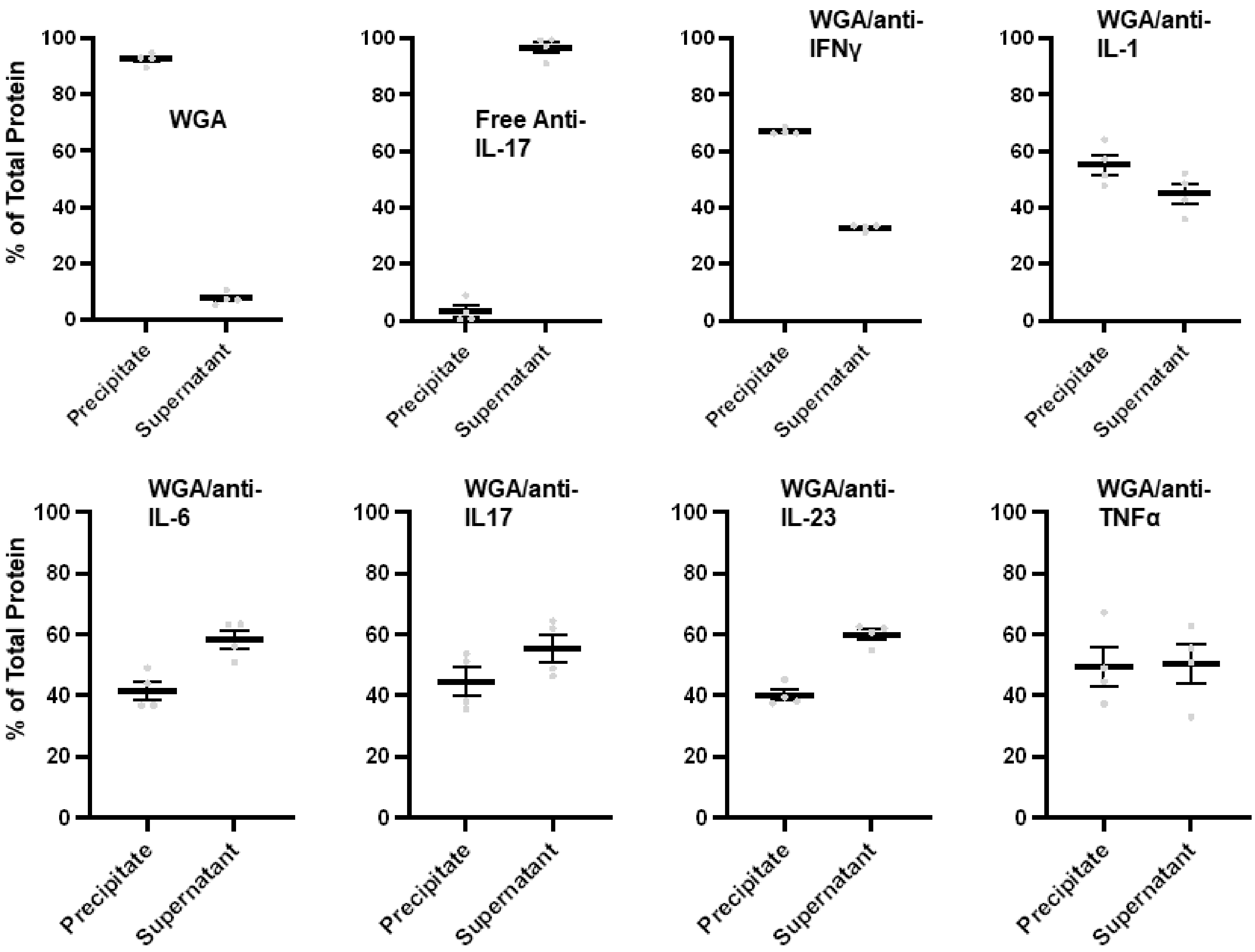

Supplementary Fig. 3. Efficiency of conjugation. The amounts of protein bound to WGA were estimated by a pull-down assay with GlcNAc beads. The precipitates represent the % of protein that binds to GlcNAc. Means ± SD of quadruplicate determinations are shown.

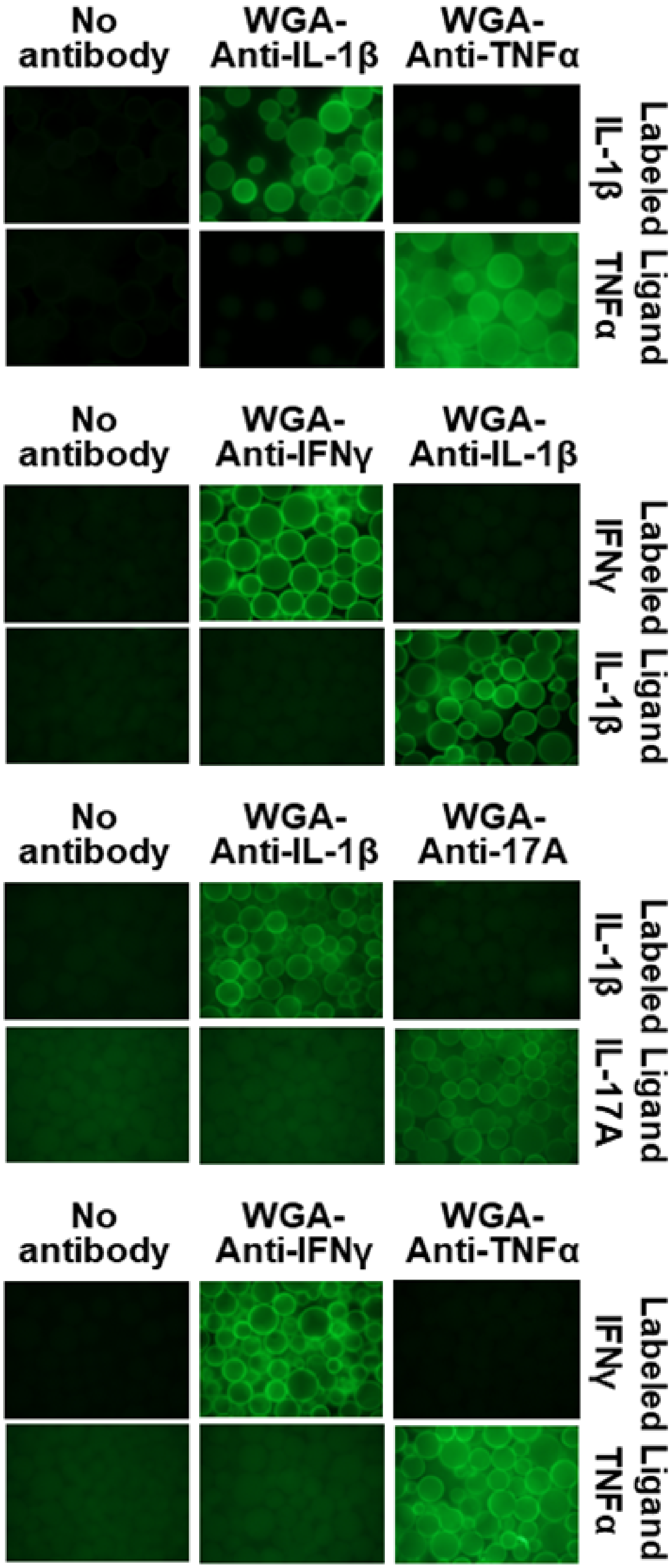
No antibody
WGA-Anti-IL-1β
WGA-Anti-TNFα
Labeled Ligand
IL-1β
TNFα
No antibody
WGA-Anti-IFNγ
WGA-Anti-IL-1β
Labeled Ligand
IFNγ
IL-1β
No antibody
WGA-Anti-IL-1β
WGA-Anti-17A
Labeled Ligand
IL-1β
IL-17A
No antibody
WGA-Anti-IFNγ
WGA-Anti-TNFα
Labeled Ligand
IFNγ
TNFα

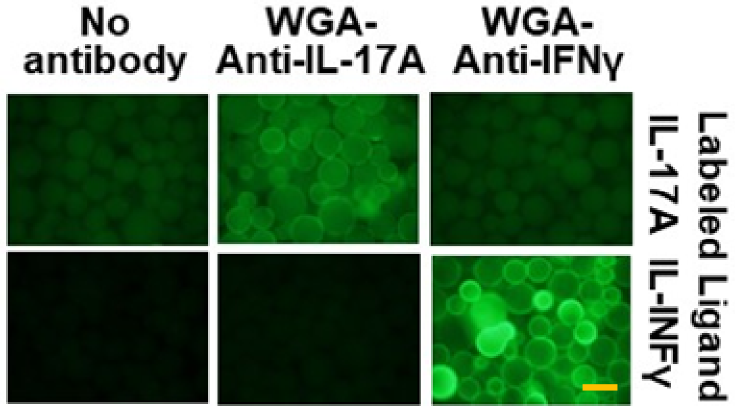

Supplementary Fig. 4. Preservation of binding specificity in WGA/antibody complexes. WGA was conjugated to the indicated antibodies and incubated with the specified labeled ligands. All tested combinations of the complexes recognize their cognate ligands, but not unrelated ligands. Binding does not occur to the WGA anchor, which is present in all the WGA/antibody complexes. The orange scale bar represents 0.1 cm.

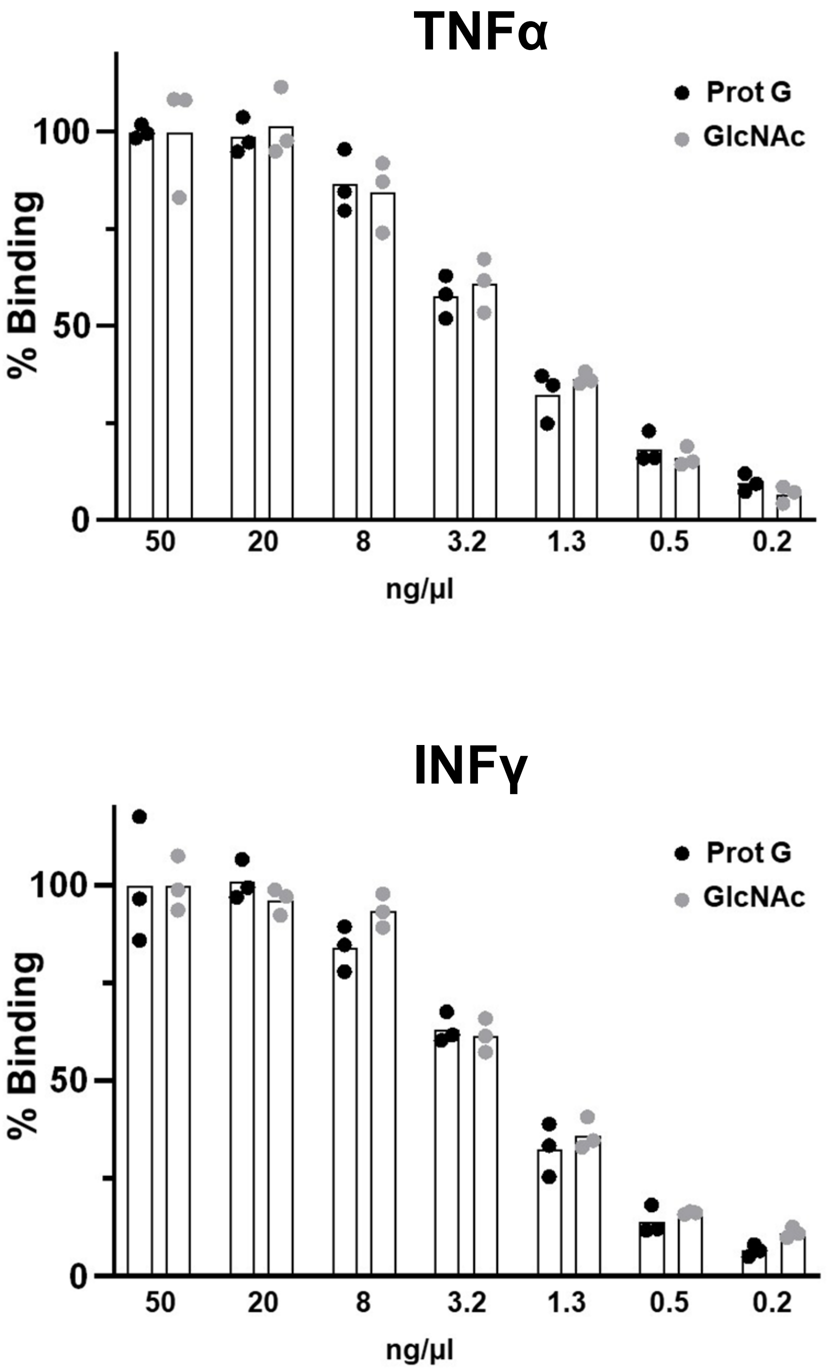

Supplementary Fig. 5. Titration of antibody binding. Free antibodies were bound to Protein G beads or WGA/antibody complexes were bound to GlcNAc beads, and they were then incubated with AlexaFluor-labeled ligands. Protein G binds at a location far from the antigen binding sites in IgG and does not interfere with antigen binding[1]. Means of triplicate determinations are shown.

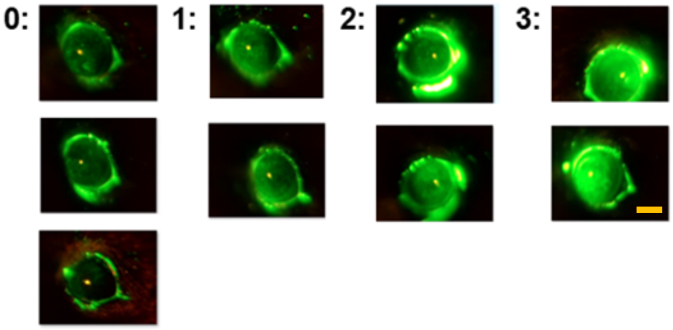

Supplementary Fig. 6. Calibration scale for fluorescein staining of eyes. The orange scale bar represents 0.5 cm.

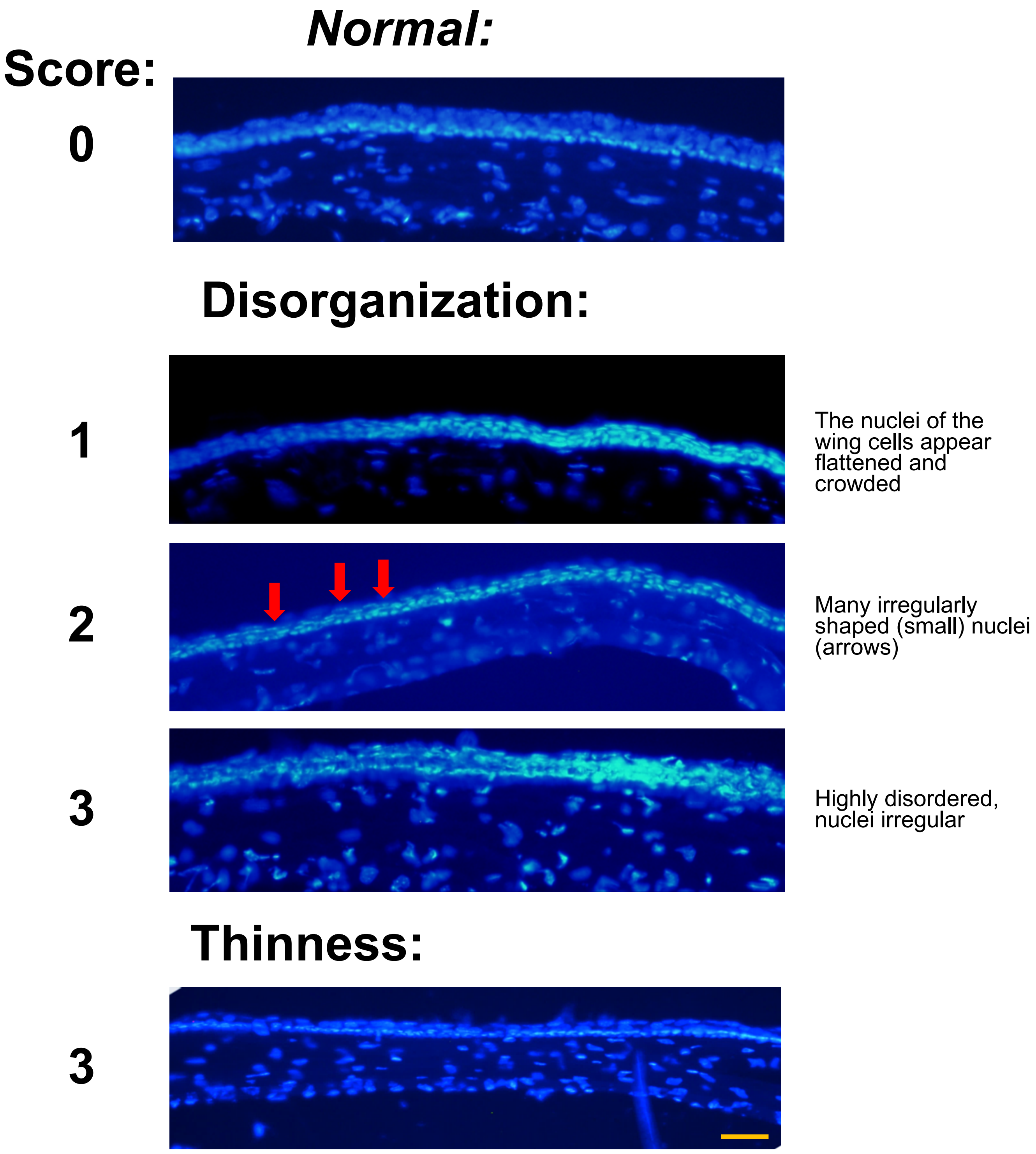

Supplementary Fig. 7. Calibration scale for disorganization and thickness. The orange scale bar represents 0.05 cm.

Reference

1. Derrick, J.P. & Wigley, D.B. The third IgG-binding domain from streptococcal protein G. An analysis by X-ray crystallography of the structure alone and in a complex with Fab. *J Mol Biol* **243**, 906-918 (1994).
