# Supplementary material for: Extending the use of biologics to mucous membranes by attachment of a binding domain
Source: Commun Biol. 2023 May 2;6:477. doi: 10.1038/s42003-023-04801-6 (PMC10154311; doi:10.1038/s42003-023-04801-6)
Supplement: Supplementary file 3 — Description of Additional Supplementary Files [file 42003_2023_4801_MOESM3_ESM.pdf]

Description of Additional Supplementary Files

File name: Supplementary Data 1

Description: Data underlying the figures
