# Supplementary material for: Extending the use of biologics to mucous membranes by attachment of a binding domain
Source: Commun Biol. 2023 May 2;6:477. doi: 10.1038/s42003-023-04801-6 (PMC10154311; doi:10.1038/s42003-023-04801-6)
Supplement: Supplementary file 4 — Supplementary Data 1 [file 42003_2023_4801_MOESM4_ESM.docx]

**EXTENDING THE USE OF BIOLOGICS TO MUCOUS MEMBRANES BY ATTACHMENT OF A BINDING DOMAIN**

**Robert M. Q. Shanks, Eric G. Romanowski, John E. Romanowski, Katherine Davoli, Nancy A. McNamara, and Jes K. Klarlund**

**Data Used in Graphs**

**Fig. 3**

**Fig. 4**

**Fig. 5**

**Fig. 6**

**Supplementary Fig. 3**

**Supplementary Fig. 5**
