# Supplementary material for: Extending the use of biologics to mucous membranes by attachment of a binding domain
Source: Commun Biol. 2023 May 2;6:477. doi: 10.1038/s42003-023-04801-6 (PMC10154311; doi:10.1038/s42003-023-04801-6)
Supplement: Supplementary file 5 — Reporting Summary [file 42003_2023_4801_MOESM5_ESM.pdf]

nature portfolio

Corresponding author(s): Jes K Klarlund

Last updated by author(s): 21/3/2023

Reporting Summary

Nature Portfolio wishes to improve the reproducibility of the work that we publish. This form provides structure for consistency and transparency in reporting. For further information on Nature Portfolio policies, see our Editorial Policies and the Editorial Policy Checklist.

Statistics

For all statistical analyses, confirm that the following items are present in the figure legend, table legend, main text, or Methods section.

n/a	Confirmed	
☐	☒	The exact sample size (*n*) for each experimental group/condition, given as a discrete number and unit of measurement
☐	☒	A statement on whether measurements were taken from distinct samples or whether the same sample was measured repeatedly
☐	☒	The statistical test(s) used AND whether they are one- or two-sided<br>*Only common tests should be described solely by name; describe more complex techniques in the Methods section.*
☒	☐	A description of all covariates tested
☐	☒	A description of any assumptions or corrections, such as tests of normality and adjustment for multiple comparisons
☐	☒	A full description of the statistical parameters including central tendency (e.g. means) or other basic estimates (e.g. regression coefficient) AND variation (e.g. standard deviation) or associated estimates of uncertainty (e.g. confidence intervals)
☐	☒	For null hypothesis testing, the test statistic (e.g. *F*, *t*, *r*) with confidence intervals, effect sizes, degrees of freedom and *P* value noted<br>*Give P values as exact values whenever suitable.*
☒	☐	For Bayesian analysis, information on the choice of priors and Markov chain Monte Carlo settings
☒	☐	For hierarchical and complex designs, identification of the appropriate level for tests and full reporting of outcomes
☒	☐	Estimates of effect sizes (e.g. Cohen's *d*, Pearson's *r*), indicating how they were calculated

Our web collection on statistics for biologists contains articles on many of the points above.

Software and code

Policy information about availability of computer code

Data collection	N/A
Data analysis	N/A

For manuscripts utilizing custom algorithms or software that are central to the research but not yet described in published literature, software must be made available to editors and reviewers. We strongly encourage code deposition in a community repository (e.g. GitHub). See the Nature Portfolio guidelines for submitting code & software for further information.

Data

Policy information about availability of data

All manuscripts must include a data availability statement. This statement should provide the following information, where applicable:

- Accession codes, unique identifiers, or web links for publicly available datasets
- A description of any restrictions on data availability
- For clinical datasets or third party data, please ensure that the statement adheres to our policy

Data underlying the figures are in the Supplementary Information. Other data generated and analyzed during the current study are available from the corresponding author upon reasonable request.

Human research participants

Policy information about studies involving human research participants and Sex and Gender in Research.

Reporting on sex and gender	N/A
Population characteristics	N/A
Recruitment	N/A
Ethics oversight	N/A

Note that full information on the approval of the study protocol must also be provided in the manuscript.

Field-specific reporting

Please select the one below that is the best fit for your research. If you are not sure, read the appropriate sections before making your selection.

☒ Life sciences ☐ Behavioural & social sciences ☐ Ecological, evolutionary & environmental sciences

For a reference copy of the document with all sections, see nature.com/documents/nr-reporting-summary-flat.pdf

Life sciences study design

All studies must disclose on these points even when the disclosure is negative.

Sample size	The Institutional Animal Care and Use Committee (IACUC) at the University of Pittsburgh requires power analysis of all experiments involving animals and performs a rigorous review before any experimentation is approved. Group sizes were calculated using α=0.05, and a power of 0.85 using the G*Power program (University of Dusseldorf).
Data exclusions	Eyes with corneal ulcers were excluded. Ulcers are very obvious macroscopically, by fluorescien staining, and upon histological examination. This led to exclusion of 0-11% of the eyes in different experiments.
Replication	Binding of labeled ligands was performed for all preparations of anchored antibodies (as in Fig. 2), confirming that conjugation worked in every case. During establishment and optimization of the conjugation procedure and the animal model >10 experiments were performed to confirm enhanced retention of antibodies that all showed very large increased of retention upon attachment of wheat germ agglutinin. Histological localization of applied anchored vs. unanchored was performed > 5 experiments with consistent results. Efficacy studies were performed at least twice with each of the tested antibodies, with consistent results. The data on histological markers (Fig. 5) was compiled from two separate experiments.
Randomization	After operation and before scoring, the mice were randomized between cages. This was simply done by placing them in a bucket, and distributing them among the different cages (5 in each).
Blinding	Photographs of fluorescein stained eyes were scored before start of treatments by two masked observers, and after treatment by the same observers as described in detail in the Methods section. Histological samples were scored by two masked observers as described in the Methods section.

Reporting for specific materials, systems and methods

We require information from authors about some types of materials, experimental systems and methods used in many studies. Here, indicate whether each material, system or method listed is relevant to your study. If you are not sure if a list item applies to your research, read the appropriate section before selecting a response.

Materials & experimental systems

n/a	Involved in the study
☐	☒ Antibodies
☒	☐ Eukaryotic cell lines
☒	☐ Palaeontology and archaeology
☐	☒ Animals and other organisms
☒	☐ Clinical data
☒	☐ Dual use research of concern

Methods

n/a	Involved in the study
☒	☐ ChIP-seq
☒	☐ Flow cytometry
☒	☐ MRI-based neuroimaging

Antibodies

Antibodies used	For treatments:<br>anti-IL-1β, clone 1400.24.17 (Invitrogen) Fisher MM425B, lot UH29071B<br>anti-IL-6, clone MP5 20F3 (R&D Biosystems), MAB406, lot: AHV2316121<br>anti-IL-17A, clone TC11-18H10 (BD Biosciences) Fisher 555068, lots 7285967, 8173732, 8141657, 9014513<br>anti-IL-23, clone G23-8 (Invitrogen) Fisher 16-7232-85, lot: 2014042<br>anti-IL-INFγ, clone H22 (R&D Bioystems) Fisher MAB4851, lot BKX1415021<br>anti-TNFα, clone XT22 (Invitrogen). Fisher MM350D, lots QC211491, TA265435<br>For immunohistochemistry:<br>Anti-CD4, clone GK1.5 (Invitrogen) Fisher 50-0041-82, lot1931458<br>Anti-beta-3 tubulin, clone 2G10-TB3 (Invitrogen) Fisher 50-4510-80, lot 2266065<br>Anti rat IgG (Invitrogen) Fisher A21094, lot 2087716
Validation	Validation of the antibodies used for treatment of mice is described in Fig. 2 in the manuscript by directly verifying binding to their cognate ligands. See also Supplemental Fig. S2. Validation of the anti CD4 antibody relied on the observation that a different antibody (clone 4SM95) labeled with a different fluorophore labeled the same cells. The anti tubulin antibody stained nerve fibers that have a very characteristic morphology. The anti rat IgG antibody was validated by the observation that strong signals were only seen in corneas treated with WGA-anti IL17 (which was raised in a rat), but not in untreated corneas (Fig. 3).

Animals and other research organisms

Policy information about studies involving animals; ARRIVE guidelines recommended for reporting animal research, and Sex and Gender in Research

Laboratory animals	8-10 week old female C57BL/6 mice (Charles River Laboratories) were used
Wild animals	N/A
Reporting on sex	Dry eye especially affects older women, which was a primary reason to use female mice. In addition, we found that fighting among male mice resulted in frequent scratches and other damages to corneas, which complicated analysis.
Field-collected samples	N/A
Ethics oversight	N/A

Note that full information on the approval of the study protocol must also be provided in the manuscript.
