# Supplementary material for: Extending the use of biologics to mucous membranes by attachment of a binding domain
Source: Commun Biol. 2023 May 2;6:477. doi: 10.1038/s42003-023-04801-6 (PMC10154311; doi:10.1038/s42003-023-04801-6)
Supplement: Supplementary file 1 — Klarlund_Peer Review File [file 42003_2023_4801_MOESM1_ESM.pdf]

nature portfolio

Peer Review File

Overcoming Washout of Biologics at Mucous Membranes After Topical Application.

Open Access This file is licensed under a Creative Commons Attribution 4.0 International License, which permits use, sharing, adaptation, distribution and reproduction in any medium or format, as long as you give appropriate credit to the original author(s) and the source, provide a link to the Creative Commons license, and indicate if changes were made. In the cases where the authors are anonymous, such as is the case for the reports of anonymous peer reviewers, author attribution should be to 'Anonymous Referee' followed by a clear attribution to the source work. The images or other third party material in this file are included in the article's Creative Commons license, unless indicated otherwise in a credit line to the material. If material is not included in the article's Creative Commons license and your intended use is not permitted by statutory regulation or exceeds the permitted use, you will need to obtain permission directly from the copyright holder. To view a copy of this license, visit http://creativecommons.org/licenses/by/4.0/.

Reviewers' comments:

Reviewer #1 (Remarks to the Author):

Several infectious and non-infectious diseases affect the ocular surface. In the Covid-19 pandemic, ocular surface mucosa has also been implicated as a potential route of viral entry. Current treatment for ocular surface diseases involves the topical application of therapeutic modalities. However, several innate immune components such as tear film, eye blinking, etc. reduce the retention of drugs at the ocular surface causing frequent use of eye drops. Often patients don't adhere to this clinical practice of frequent eye drops, impairing the treatment. Therefore, there is an unmet need to develop strategies for better retention of drugs at the ocular surface. In this proof-of-concept study, authors Shanks et al evaluated the benefits of WGA-conjugated antibodies as a topical treatment alternative for dry eyes. The authors have shown the retention ability of the drug on the mucous membrane as well as the efficacy of anchored antibodies to protect corneal health in a mouse model. Overall, the manuscript is very well written and the study design is very robust with well-executed experiments. The study has high translational value in bringing the research from the bench to the bedside. This reviewer has the following minor comments.

Minor comments:

1) The authors mentioned intraperitoneal administration of antibodies to IL-17A has been shown to reduce signs of dry eyes. How authors envision direct comparison of neutralizing antibodies and their effects on reducing dry eyes when administered intraperitoneally vs. topical (with WGA and without WGA).
2) According to the data, WGA conjugated antibodies were retained longer with a 350-fold higher half-life. Did the authors check if there was any problem with visual acuity or blurred vision as the compound was persistently attached for a long span of time, which might be a side-effect in the clinical scenario? An experiment to show any non-toxic effect will be an excellent addition.
3) Figure 1 panel A, should be αIFN-γ and not αIL-INFγ. Also, several other places where IFN-γ has been written as INFγ. Please change.
4) Please include scale bars for fluorescence imaging. (Fig 3, Fig 5)
5) Fig 5, please mention the number of days for antibody treatment before the corneas were harvested for staining.
6) Please include the number of samples (n=) in each figure legend for the respective study.
7) Line 163, instead of 'prevented' the authors can use the term 'rescued the loss of sub-epithelium nerve plexus' or 'a further loss was prevented' as the conjugated antibodies were given post dry eye pathologies and the loss of nerve plexus already started happening.
8) Line 169: Fluorescein staining was shown in Figure 4 and not in 3A. Please clarify.
9) Line 186: rephrase. Can be written as 'despite their great potency"

Reviewer #2 (Remarks to the Author):

The article submitted to Communications Biology by Shanks et al., describe a facile strategy to conjugate wheat germ agglutinin to antibodies and establish the efficacy in mice model of dry eye treatment. The poor bioavailability of biologics at the ocular surface is one of the several reasons that warrant alternative drug delivery methods. The authors used a simple strategy of conjugating wheat germ agglutinin to antibodies to increase the residence time of the later. Using fluorescently labelled probes the authors establish that the conjugated antibodies has about 350 fold higher retention and tissue penetration than the unconjugated forms. The authors further confirm the efficacy of the conjugated antibodies in a mice model of severe dry eye. Overall, the manuscript is well-written and the increased retention and efficacy of conjugated are demonstrated convincingly. Therefore, I recommend this manuscript for publication in Communcation Biology after Minor comments as outlined below.

Minor Comments
1. The authors used SDS-PAGE to demonstrate the facile conjugation of anti-17A antibody to WGA.

However, the authors did not determine purity/homogeneity of the preparations. For example, in the SDS-PAGE (Fig. S1) there are numerous additional bands that are not accounted for.
2. In the mice models of dry eye, the authors tested the efficacy of 6 other antibodies and only 3 turned out be effective. The authors mentioned that insufficient use of antibodies could be responsible for this. However, this not convincing because the concentration used by the authors is sufficiently high enough to bind to the cytokines. This discrepancy has to be addressed in the revised version.
3. Protocols for SDS-PAGE was not included in the experimental protocol.

Reviewer #3 (Remarks to the Author):

This paper examines conjugation of wheat germ agglutinin (WGA) to anti-cytokine antibodies in order to decrease loss due to washout in an animal model of dry eye disease. The data presented is encouraging for the approach as a general method to increase the half-life of topically administered biologics in the cornea. My main criticisms are the characterization of the antibody conjugates, their effect on antibody binding kinetics, and what appears to be significant background binding in an in vitro bead-based assay. This may bleed into the authors' later observations regarding the efficacy of some conjugated antibodies in treating dry eye, and undermine some of the paper's robustness in terms of reproducibility. In addition, the paper relies heavily on scoring as a measure. Although unavoidable in the assessment of dry eye, and measures seem to have been taken to ensure the fairness and consistency, more transparency with the raw data and score would serve to give the reader more confidence in the results. The article presents some negative data, which I think is healthy, but perhaps requires a more detailed characterization of their generated reagents, and more transparent reporting of their results.

1. Introduction: it would be good if the authors could mention the context for this conjugation strategy in the introduction. Conjugation is a frequently employed tactic to alter biologic pharmacokinetics, is this the first example in the eye? There is some mention in the discussion, but it might be more helpful in the introduction to give the reader context.

2. Fig. S1: SDS-PAGE is used to confirm labeling of antibodies with WGA following EDC/NHS activation. It would be helpful to see the gels for each antibody labeled rather than just one with the generic "Ab" label. For instance, as a reader, one might wonder if the labeling is different in the antibodies that don't work in the subsequent in vivo assay.

3. Fig. S1: It appears that in the "Ab+WGA*" sample there is streaking and more bands than the two additional conjugated heavy and light chain bands that are expected. This is despite the total protein concentration appearing lower. Does conjugation with WGA affect the stability of the antibodies? Can the authors comment on the reasons for the additional bands?

4. Fig. S1: It appears that much of the antibody is unlabeled after the conjugation, was any additional purification performed besides centrifugal filtration? Does the labeling efficiency hint that there are problems with some of the antibody molecules that cause them to go unlabeled?

5. Fig. S1: When running SDS-PAGE gels of antibodies, it is helpful to show non-reducing conditions alongside reducing conditions so that the antibody quality can be better assessed. In this case, it is important in order to gauge the stoichiometry of labeling. Are most antibodies labeled once on either heavy or light chain (1:1 stoichiometry)? Or are the antibodies that are labeled labeled on both heavy and light chains (2:1 stoichiometry)? This information is lost under reducing conditions.

6. Fig. S1: Do these gels show the antibodies prior to or after purification? SDS-PAGE could be an important part of analyzing the quality of protein after conjugation and purification, prior to any assays.

7. Fig. 2A: There appears to be significant background binding in the αIL-6, αIL-17, and αTNFα

samples. This potentially indicates that the ligand concentrations are too high, or that the washing is insufficient. (Activated and purified) Anchor alone could also be an important control to add to the experiment.

8. Fig. 2A: To be convincing, it would be best to quantify the binding and demonstrate a titration response. The question being asked is whether conjugation affects binding (of either WGA or the antibody), but a more important one is, perhaps, how conjugation affects affinity of the antibody. In these, I assume, saturation binding experiments, it is difficult to measure how the antibody's activity has been affected by conjugation.

9. Fig. 2B: The competition assay goes some of the way to addressing my previous point, although, again, a titration is important to show convincing evidence of binding and competition.

10. Fig. 2B: Was the competition experiment repeated for all antibodies? It would be good to see this data in the supplement.

11. Fig. 2B: The "no competitor" sample seems a lot brighter than in Fig. 2A for the αIL-1β antibody. Are the conditions the same?

12. Fig. S2: It is good to see that the authors tried different combinations of antibody and antigen, however it would be more helpful to see all (or more) combinations of antibodies with all antigens. Particularly those of IL-18 and IL-6 which may be prone to background binding.

13. Fig. 3: Can the authors mention the number of mice/eyes used for each experiment in the figure legends?

14. Fig. 3A: Can the authors comment on why the error bars are so much bigger in the anchor sample? If the number of eyes is small, would it be possible to have a plot with the raw data?

15. Fig. 3B: Preincubation with Rat anti-Mouse IgG is good.

16. Fig. 3C: Can the authors comment on why after 24 hours the WGA-conjugated antibody has concentrated between the epithelium and Bowman's membrane? Is it eventually cleared? Does repeated administration lead to accumulation at this site? Is GlcNac concentration higher here?

17. Application of anchored anti IL-17A antibody once a day is sufficient to treat dry eye: It is mentioned that choosing IL-17A as a target because IP administration of αIL-17 antibody was shown to help with dry eye. Did the authors compare their antibody conjugate with IP administration of αIL-17 antibody? This is perhaps the more medically meaningful comparison. Though I appreciate that efficacy by topical administration is, in and of itself, significant.

18. Application of anchored anti IL-17A antibody once a day is sufficient to treat dry eye: Did the authors try more frequent dosing of the unconjugated antibody? Is it known how frequently the unconjugated antibody would need to be dosed to see an effect? Can the concentration of the unconjugated antibody simply be increased?

19. 147-148: Can the authors comment on why day 20 was chosen to discontinue treatment? Do the effects plateau here?

20. Fig. 4: Could a supplemental figure showing the score for each mouse along with the summary statistics be shown?

21. Fig. 4: It appears that in the mice treated with no anchor, after day 30 the staining begins to decrease, is this due to deterioration of the epithelium supporting accumulation of the unconjugated antibodies?

22. Fig. 5 and 6: Could the authors overlay the raw data/scores on the dynamite plots?

23. 179--183 and 212--215: Can the authors comment on whether these targets should be

expected to treat dry eye disease? Was this unexpected?

24. 212--215: Was any IHC done for treatments that didn't improve?

25. 212--215: Perhaps the authors can add to their point on concentration that conjugation may negatively affect affinity (which wouldn't be detected in their qualitative binding assay), which could explain their negative results.

26. 249--266: Was the NHS/EDC coupling reaction quenched before purification?

Response to Reviewers

We thank the reviewers for their helpful critical comments. Each point was addressed and listed below. We think that the suggestions have improved the text and have supported and expanded the findings in the manuscript.

Reviewer 1	
1) The authors mentioned intraperitoneal administration of antibodies to IL-17A has been shown to reduce signs of dry eyes. How authors envision direct comparison of neutralizing antibodies and their effects on reducing dry eyes when administered intraperitoneally vs. topical (with WGA and without WGA).	We are certainly very interested in developing this new delivery method further, and we anticipate analyzing different dosages and frequencies of application, comparing routes of administration (including intraperitoneal delivery), and studying different anchors. We expect that patients would rather use topical drops than have intraperitoneal injections of antibodies, so are focusing on the topical application as a primary modality. However, the intraperitoneal injection study definitely helped us to choose IL-17A as a candidate. While the question brings up potential differences in mechanism, this study is more translationally oriented.
2) According to the data, WGA conjugated antibodies were retained longer with a 350-fold higher half-life. Did the authors check if there was any problem with visual acuity or blurred vision as the compound was persistently attached for a long span of time, which might be a side-effect in the clinical scenario? An experiment to show any non-toxic effect will be an excellent addition.	A prior report found no negative effect of WGA on visual acuity, WGA has been found to exhibit low toxicity in rabbit and human eyes, and fluorescein labeled WGA has been suggested as a routine tool to detect dry eye (PMID: 31451311, 20823935). This is important and has now been noted in the text (lines 248-251).
3) Figure 1 panel A, should be αIFN-γ and not αIL-INFγ. Also, several other places where IFN-γ has been written as INFγ. Please change.	This has been changed.
4) Please include scale bars for fluorescence imaging. (Fig 3, Fig 5)	This has been done. Please see legends.
5) Fig 5, please mention the number of days for antibody treatment before the corneas were harvested for staining.	This has been done. Please see the legend.
6) Please include the number of samples (n=) in each figure legend for the respective study.	This has been done.
7) Line 163, instead of ‘prevented’ the authors can use the term ‘rescued the loss of sub-epithelium nerve plexus’ or ‘a further loss was prevented’ as the conjugated antibodies were given post dry eye pathologies and the loss of nerve plexus already started happening.	This has been done. Please see line 179.
8) Line 169: Fluorescein staining was shown in Figure 4 and not in 3A. Please clarify.	The error has been corrected.
9) Line 186: rephrase. Can be written as ‘despite their great potency”	We agree. This has been changed.

<table>
<tr><td colspan="2">Reviewer 2</td></tr>
<tr><td>1. The authors used SDS-PAGE to demonstrate the facile conjugation of anti-17A antibody to WGA. However, the authors did not determine purity/homogeneity of the preparations. For example, in the SDS-PAGE (Fig. S1) there are numerous additional bands that are not accounted for.</td><td>Please see response to question 3 from reviewer 3.</td></tr>
<tr><td>2. In the mice models of dry eye, the authors tested the efficacy of 6 other antibodies and only 3 turned out be effective. The authors mentioned that insufficient use of antibodies could be responsible for this. However, this not convincing because the concentration used by the authors is sufficiently high enough to bind to the cytokines. This discrepancy has to be addressed in the revised version.</td><td>We agree that the problem is probably not simply that insufficient amounts were used. However, we are hesitant to draw firm conclusions from these negative data. The situation in vivo is complicated, the localization and kinetics of production of endogenous cytokines could preclude efficient neutralization by the antibodies. We have added text to the discussion to address this important issue. Please see lines 237-242 in the revised manuscript.</td></tr>
<tr><td>3. Protocols for SDS-PAGE was not included in the experimental protocol.</td><td>Included now. Please see lines 299-305.</td></tr>
<tr><td colspan="2">Reviewer 3</td></tr>
<tr><td>1. Introduction: it would be good if the authors could mention the context for this conjugation strategy in the introduction. Conjugation is a frequently employed tactic to alter biologic pharmacokinetics, is this the first example in the eye? There is some mention in the discussion, but it might be more helpful in the introduction to give the reader context.</td><td>Covalent conjugation has indeed been used frequently to attach polyethylene glycol to improve pharmacokinetics by increasing half-lives in the bloodstream, and FDA approved drugs are available (see https://www.biochempeg.com/article/58.html)
A limited number of reports describe attachment of modules that immobilize drugs which prolongs residency after injection into tissues (see references 15 and 16). The thrust of this paper is to demonstrate that WGA conjugated antibody biologics can be used topically even in the presence of a very effective clearance mechanism in the eye.
We have now mentioned this in the introduction. Please see lines 84-95.</td></tr>
<tr><td>2. Fig. S1: SDS-PAGE is used to confirm labeling of antibodies with WGA following EDC/NHS activation. It would be helpful to see the gels for each antibody labeled rather than just one with the generic "Ab" label. For instance, as a reader, one might wonder if the labeling is different in the antibodies that don't work in the subsequent in vivo assay.</td><td>The conjugation efficiency is indeed an important parameter. Due to the heterogeneity of the reaction products (see reply to comment 3) it is difficult to quantitate gels accurately. Instead, we have devised an assay to obtain quantitative labeling efficiency which we now show in Fig S3. Of note, the efficiencies of conjugation of the antibodies were reasonably similar, and there was no correlation between level of conjugation and in vivo effect on fluorescein staining. The antibody shown in Supplemental Fig. 1 is anti-IL-17A, which is now noted in the legend.</td></tr>
<tr><td>3. Fig. S1: It appears that in the "Ab+WGA*" sample there is streaking and more bands than the two additional conjugated heavy and light chain bands that are expected. This is despite the total protein concentration appearing lower. Does conjugation with WGA affect the stability of</td><td>Crosslinking is principally a random process. We devised our methodology as a two-step procedure that only activates the WGA, but not the IgG, which reduces the possible cross-links that can be formed. Still, the WGA monomer contains 6 asp, 6 glu and one C-terminal carboxy group that can be activated with EDC/NHS (according to the Uniprot database). An antibody contains more than 20 amino groups available for</td></tr>
</table>

the antibodies? Can the authors comment on the reasons for the additional bands?	conjugation (McCombs, j. and Owen, S.C. PMID: 25604608). Conjugation can occur between or within the subunits on WGA, which is a dimer, and/or to one or more positions in one or more IgG or WGA molecules. Thus, an enormous number of molecular species can be generated, many of which are branched or even circular. Binding of SDS depends, of course on the molecular weight of proteins, but their primary structure also influences their migration in gels. It is our experience that crosslinking always results in multiple bands and smears. This is also evident in other publications, see, for instance Fig. 1 in Ido *et al.* (PMID: 24914208) and Fig. 1 in Liu *et al*. (PMID: 22031695).
4. Fig. S1: It appears that much of the antibody is unlabeled after the conjugation, was any additional purification performed besides centrifugal filtration? Does the labeling efficiency hint that there are problems with some of the antibody molecules that cause them to go unlabeled?	In practice one never obtains 100% conjugation efficiency unless extreme conditions are used (high concentrations of protein and crosslinker, which yields a gel not unlike gelatin). We do not have a precedent to postulate that the antibody molecules that are not conjugated are damaged. No further purification was done to minimize losses in this proof-of-concept study. There are unconjugated antibodies in the preparations, but these unconjugated antibodies do not reduce manifestation of dry eye, as shown by the data presented in this paper. As this process is improved, enhancing conjugation efficiency and purification of conjugated antibodies is a clear goal.
5. Fig. S1: When running SDS-PAGE gels of antibodies, it is helpful to show non-reducing conditions alongside reducing conditions so that the antibody quality can be better assessed. In this case, it is important in order to gauge the stoichiometry of labeling. Are most antibodies labeled once on either heavy or light chain (1:1 stoichiometry)? Or are the antibodies that are labeled labeled on both heavy and light chains (2:1 stoichiometry)? This information is lost under reducing conditions.	We agree in principle that the improvement of conjugation efficiency and stoichiometry is important to develop this as a therapeutic for patients. We are unaware of any reason that antibodies conjugated to one WGA molecule would be preferentially conjugated with another WGA molecule, so we think most are attached to one WGA molecule, although some may be conjugated to two or more WGAs. In any case, they will have the ability to bind to sialic acid or GlcNAc whether they are attached to one or more WGA molecules (each WGA molecule has four high affinity binding sites for the ligands). The expected heterogeneity of the samples is now noted in the text, lines 258-261
6. Fig. S1: Do these gels show the antibodies prior to or after purification? SDS-PAGE could be an important part of analyzing the quality of protein after conjugation and purification, prior to any assays.	The samples were WGA or IgG loaded directly or the conjugates after purification in the 100 kDa molecular weight cut-off spin filters. Note that WGA is a dimer and it stains heavily with Coomassie blue, so a band at ~20 kDa shows up.
7. Fig. 2A: There appears to be significant background binding in the αIL-6, αIL-17, and αTNFα samples. This potentially indicates that the ligand concentrations are too high, or that the washing is insufficient. (Activated and purified) Anchor alone could also be an important control to add to the experiment.	It is not surprising that the "stickiness" of the cytokines differs and we have seeked to reduce background binding. Recently, we found that including 0.5% Triton X-100 and 1% bovine serum albumin results in some improvement, and this is now described in the Methods section (lines 326-327). The stickiness of some of the ligands limits the analyses that can be performed because a good signal/noise ratio is required (for example the experiments suggested in comments 10 and 12).

	The WGA conjugated antibodies do not bind to the WGA anchor, which is evident from the data shown in Fig. S4 in the Supplementary Information and is now also noted in the legend. We agree that the point is important and is now also noted in the legend to Fig. 2 in the manuscript.
8. Fig. 2A: To be convincing, it would be best to quantify the binding and demonstrate a titration response. The question being asked is whether conjugation affects binding (of either WGA or the antibody), but a more important one is, perhaps, how conjugation affects affinity of the antibody. In these, I assume, saturation binding experiments, it is difficult to measure how the antibody's activity has been affected by conjugation.	We agree that tritration of antibody binding is an important characterization of the conjugation procedure, and to address this we added new data in Fig. S5. We tested two antibodies that had no therapeutic efficacy, and found no significant reduction in affinities for their ligands. Reduction in their affinities upon conjugation is therefore not a likely explanation for their lack of efficacy. Text was added to describe this new data (Lines 120-121)
9. Fig. 2B: The competition assay goes some of the way to addressing my previous point, although, again, a titration is important to show convincing evidence of binding and competition.	Please see Fig. S5 and the reply to the previous comment.
10. Fig. 2B: Was the competition experiment repeated for all antibodies? It would be good to see this data in the supplement.	We agree and show additional competition experiments in Supplemental Fig. S2. All tested conjugates were competed with GlcNAc.
11. Fig. 2B: The "no competitor" sample seems a lot brighter than in Fig. 2A for the αIL-1β antibody. Are the conditions the same?	Panels 2A and 2B were done at different times with different preparations of labeled ligands and exposure times for photography. They are therefore not directly comparable. However, the exposure time was always maintained within in an experimental group. That is, for anti-IL-1β the No Ab, Ab w/o Anchor, and Ab with anchor image all had the same exposure (and the same for other antibodies).
12. Fig. S2: It is good to see that the authors tried different combinations of antibody and antigen, however it would be more helpful to see all (or more) combinations of antibodies with all antigens. Particularly those of IL-18 and IL-6 which may be prone to background binding.	We provide more examples of combinations of different antibodies and ligands (Fig. S4). The number of possible combinations is very great and we have limited the experiments to a manageable number, using ligands that produce reasonably low backgrounds (cf. comment 7). None of the tested combinations bound to the "wrong" antibody.
13. Fig. 3: Can the authors mention the number of mice/eyes used for each experiment in the figure legends?	Agreed. We have done this and it is also required be in compliance with the style of Communication Biology.
14. Fig. 3A: Can the authors comment on why the error bars are so much bigger in the anchor sample? If the number of eyes is small, would it be possible to have a plot with the raw data?	We don't really know. Different eyes do have slightly different rates of release of the conjugate. We could speculate that the levels of inflammation in the eyes probably differ and that could have an effect? But without understanding the mechanism it should perhaps not be mentioned in the text.<br>The graph (Fig 3A) has been reformatted to agree with the style of Communication Biology and now shows the individual data points.
15. Fig. 3B: Preincubation with Rat anti-Mouse IgG is good.	Thank you, we think this type of blocking experiment is the best way to measure unspecific binding and we do it routinely.
16. Fig. 3C: Can the authors comment on why after 24 hours the WGA-conjugated antibody has concentrated between the epithelium and	The exposure is not the same in panels B and C in Fig. 3 and there is not really any concentration of the conjugate. This has now been explicitly mentioned in the legend. Antibody is not

Bowman's membrane? Is it eventually cleared? Does repeated administration lead to accumulation at this site? Is GlcNac concentration higher here?	visible after 72 hours, and we now mention this in the text (Line 136). An older paper notes that the corneal basement membrane the basement membrane binds WGA strongly (Brandon *et al*. PMID: PMID: 3219860), which is now noted in the text (line 135-136). Histological examinations were only done after one application, so we cannot address on the accumulation comment.
17. Application of anchored anti IL-17A antibody once a day is sufficient to treat dry eye: It is mentioned that choosing IL-17A as a target because IP administration of αIL-17 antibody was shown to help with dry eye. Did the authors compare their antibody conjugate with IP administration of αIL-17 antibody? This is perhaps the more medically meaningful comparison. Though I appreciate that efficacy by topical administration is, in and of itself, significant.	Since we are primarily focused on a future therapeutic approach enabling the use of topical application of biologics on the ocular surface, we did not consider this comparison. However, it would indeed be of considerable interest to examine many different treatments regimens and different anchors. Please see reply to reviewer 1's point 1 about generating such data
18. Application of anchored anti IL-17A antibody once a day is sufficient to treat dry eye: Did the authors try more frequent dosing of the unconjugated antibody? Is it known how frequently the unconjugated antibody would need to be dosed to see an effect? Can the concentration of the unconjugated antibody simply be increased?	These are certainly relevant questions, but please see reply to point 17. Given the very rapid wash-out of the antibody, we would not expect that increasing the concentration would be sufficient. but it seems possible that very frequent application of antibody would be effective. The point here is, of course, to demonstrate that we can avoid frequent administration of biologics. We have been told by clinicians that requiring patients to apply drugs more than twice daily (typically morning and evening) results in sharply decreased compliance. One FDA biologic, Oxervate, is an approved biologic for use on the ocular surface, and it requires a 6x per day dosing regimen (line 216).
19. 147-148: Can the authors comment on why day 20 was chosen to discontinue treatment? Do the effects plateau here?	We have not seen any significant increase of the effects after 21 days. Accordingly, we have considered 20-28 days of treatment as sufficient to induce maximum effects for our experiments.
20. Fig. 4: Could a supplemental figure showing the score for each mouse along with the summary statistics be shown?	Fig. 4 is constructed from a total of ~300 individual measurements from 19 different eyes and is therefore not suited for graphical representation with individual data points. Instead, we present the data in a tabular form in the Supplementary information (Fig. S6). The summary statistics is now shown in Fig 4.
21. Fig. 4: It appears that in the mice treated with no anchor, after day 30 the staining begins to decrease, is this due to deterioration of the epithelium supporting accumulation of the unconjugated antibodies?	The decrease is not statistically significant in this experiment and a decrease was not noted in other experiments. At this point, we do not know the reason for the downward trend in that experiment and are not comfortable speculating on the reason given our limited information.
22. Fig. 5 and 6: Could the authors overlay the raw data/scores on the dynamite plots?	We have now changed the presentation to agree with the style of Communications Biology to show the individual points.
23. 179--183 and 212--215: Can the authors comment on whether these targets should be	IFNγ, TNFα, and IL-6 have been implicated in dry eye (reference 9), but we have no expectations of their roles in this

expected to treat dry eye disease? Was this unexpected?	particular model in our model. We now discuss this point in lines 237-242. See also reply to reviewer 2’s comment 3.
24. 212--215: Was any IHC done for treatments that didn't improve?	This is an interesting point, but was not performed in this study; we will consider doing this in future work.
25. 212--215: Perhaps the authors can add to their point on concentration that conjugation may negatively affect affinity (which wouldn't be detected in their qualitative binding assay), which could explain their negative results.	Please see reply to reviewer 2’s comment 3. The results from the titrations of the ligands (Fig. S5) do not indicate that reduced affinities explain the negative results.
26. 249--266: Was the NHS/EDC coupling reaction quenched before purification?	Yes, if the conjugates were used immediately. Otherwise, excess NHS esters were simply allowed to hydrolyze spontaneously for several days (the half-life is ~4 h at the pH used in the study https://tools.thermofisher.com/content/sfs/manuals/MAN0011309_NHS_SulfoNHS_UG.pdf). This has now been added to the Methods section (line 292-293).

Reviewers' comments:

Reviewer #1 (Remarks to the Author):

Authors have addressed all my concerns. Manuscript is significantly improved.

Reviewer #2 (Remarks to the Author):

The authors responded to my comments succinctly. I do not have any further queries. The manuscript can be accepted now.

Reviewer #3 (Remarks to the Author):

I thank the authors for their detailed response. I still have reservations about the quality of the conjugation, which I feel undermines the results, promising as they may seem. I think it also makes reproduction potentially challenging. Reponses to specific points follow:

2. I appreciate updating the labels to identify which antibody is being run on the gel, but can the authors please add the gels for the other antibodies conjugated? I think this is important to understand the purity of each of the conjugates generated.

3. The text of S1 mentions that an anti IL-17A antibody is treated with EDC/NHS, but is it the WGA that is treated?

I understand that due to the nature of chemical cross-linking it is difficult to control the species generated. However, that also makes there analysis and potential removal by purification so important. The reference given (Fig. 1 in Ido et al. (PMID: 24914208)) goes so far as to excise the bands for typsin digest and mass spectrometry while also analysing the stoichiometry. The other reference (Fig. 1 in Liu et al. (PMID: 22031695)) uses a titration of coupling reagents to select optimal conditions and monitor the appearance of unexpected bands. Although the gels in both papers exhibit smears, in both cases the authors have taken care to understand what the each band is and the stoichiometry of labeling. This is not done here.

4. It appears from figure S3 that conjugation "efficiency" is around 40 % -- 70 %, depending on the sample. In such a case, purification should be used to separate both unconjugated material and side products. Otherwise a mixture of WGA and antibody that are not conjugated, should be used as the control.

5. Reducing and non-reducing conditions for SDS-PAGE would still clear up a number of the stoichiometry and purity issues.

6. This again indicates the purification procedure used here is doing little more that dialysing the sample buffer.

7. I appreciate that it is not surprising that the "stickiness" of the cytokines differs. However, the data presented does not mitigate for this stickiness (for example but titrating the reagent, competing, or calculating binding stoichiometries). That the stickiness of some ligands limited the analysis is perhaps further evidence that another method of analysis needs to be used. Increased presence of antibodies on the beads, for instance, could provide be providing more area for a sticky ligand to bind.

Responses to Reviewer 3

We had revised the manuscript extensively in our previous submission. Revisions included competition experiments with additional antibodies (Fig. S2), and experiments demonstrating specificity of binding with different antibodies (Fig. S4). Importantly, we established that affinities are not affected by conjugation (Fig. S5), as suggested by the reviewer. We now include a paragraph discussing the limitations of the preparations (lines 269-277).

We believe that gel electrophoresis of our preparations is not very meaningful because an enormous number of different molecules are generated due to the random nature of chemical conjugation, and most of these appear as smears that are not visualized clearly on SDS gels. For this reason, we devised a new pull-down procedure so the amount of conjugation could be measured quantitively (Fig. S2). This supplements the qualitative experiments that demonstrate that the conjugated preparations contain molecules that have binding sites for both GlcNAc and for the ligands of the antibodies (Fig. 2 in the main text).

[No comment #1]	
2. I appreciate updating the labels to identify which antibody is being run on the gel, but can the authors please add the gels for the other antibodies conjugated? I think this is important to understand the purity of each of the conjugates generated.	Please see second paragraph above.
3. The text of S1 mentions that an anti IL-17A antibody is treated with EDC/NHS, but is it the WGA that is treated?	This has been corrected.
4. It appears from figure S3 that conjugation "efficiency" is around 40 % -- 70 %, depending on the sample. In such a case, purification should be used to separate both unconjugated material and side products. Otherwise a mixture of WGA and antibody that are not conjugated, should be used as the control.	We are not convinced that gel electrophoresis gives much meaningful information. As mentioned above, we therefore devised a pull-down procedure so quantitative data could be obtained. Any unconjugated antibody present is washed out very quickly, and does not have any biological effects (Figs.2 and 3). Likewise, any free WGA is biologically inactive (Fig. 6).
5. Reducing and non-reducing conditions for SDS-PAGE would still clear up a number of the stoichiometry and purity issues.	Please see second paragraph above.
6. This again indicates the purification procedure used here is doing little more that dialysing the sample buffer.	Please see reply to point 4.
7. I appreciate that it is not surprising that the "stickiness" of the cytokines differs. However, the data presented does not mitigate for this stickiness (for example but titrating the reagent, competing, or calculating binding stoichiometries). That the stickiness of some ligands limited the analysis is perhaps further	Background binding is, of course, a general problem in biochemical assays. We did optimize the conditions as mentioned in our last submission and noted this in the Methods Section. This reduced the backgrounds somewhat, and Figs. S3 - S5 were done using

evidence that another method of analysis needs to be used. Increased presence of antibodies on the beads, for instance, could provide be providing more area for a sticky ligand to bind.	the optimized conditions. Titrations with two ligands are presented in Fig. S5.
